# Emerging role of herbal nanogel formulations in skin cancer therapy: mechanistic insights and translational advances in nanomedicine

**DOI:** 10.1186/s12951-026-04788-2

**Published:** 2026-07-18

**Authors:** Suraj Kumar, Rishabha Malviya, Phool Chandra, Sathvik Belagodu Sridhar, Javedh Shareef, Tarun Wadhwa, Daniel Arockiam

**Affiliations:** 1 Department of Pharmacy, School of Medical and Allied Sciences, Galgotias University, Greater Noida, 201308 U.P India; 2 Teerthanker Mahaveer College of Pharmacy, Teerthanker Mahaveer University, Moradabad, 244001 Uttar Pradesh India; 3 College of Pharmacy, Dubai Medical University, Dubai, United Arab Emirates; 4 RAK College of Pharmacy, RAK Medical & Health Sciences University, Ras Al Khaimah, United Arab Emirates; 5 School of Engineering and IT, Manipal Academy of Higher Education, Dubai Campus, Dubai, UAE

**Keywords:** Skin cancer, Herbal nanogels, Stimuli-responsive, LHRH-targeted, Nanotechnology, Cross-linked polymers

## Abstract

**Objective:**

Skin cancer remains a major global health concern, necessitating the development of innovative therapeutic strategies that enhance treatment efficacy while minimizing adverse effects. This review aims to explore the emerging role of herbal nanogels as a promising nanotechnology-based drug delivery system for skin cancer therapy.

**Methods:**

A comprehensive review of recent literature was conducted focusing on nanogel-based drug delivery systems, herbal bioactive compounds, and stimulus-responsive nanotechnology approaches for cancer treatment, particularly skin cancer.

**Results:**

Nanogels, defined as crosslinked nanoscale polymeric networks, have gained significant attention due to their high drug-loading capacity, tunable physicochemical properties, and ability to respond to various internal and external stimuli. They can be synthesized using techniques such as emulsion methods, micro-molding, and photolithography, allowing precise control over size and morphology. Advanced stimulus-responsive nanogels, including tumor microenvironment-sensitive and ligand-targeted systems (e.g., LHRH-targeted nanogels), enable controlled and site-specific drug release. Recent developments in herbal nanogels integrate plant-derived bioactive compounds within nanogel matrices, enhancing solubility, stability, and therapeutic efficiency against skin cancer.

**Discussion:**

Herbal nanogels represent a promising interdisciplinary approach combining nanotechnology and phytomedicine, offering targeted, less invasive, and potentially more effective treatment options for skin cancer. Despite significant progress, challenges such as clinical translation, large-scale production, and long-term safety evaluation remain. Continued research in smart and multifunctional nanogel systems may significantly advance future skin cancer therapies.

**Graphical abstract:**

**Figure:**
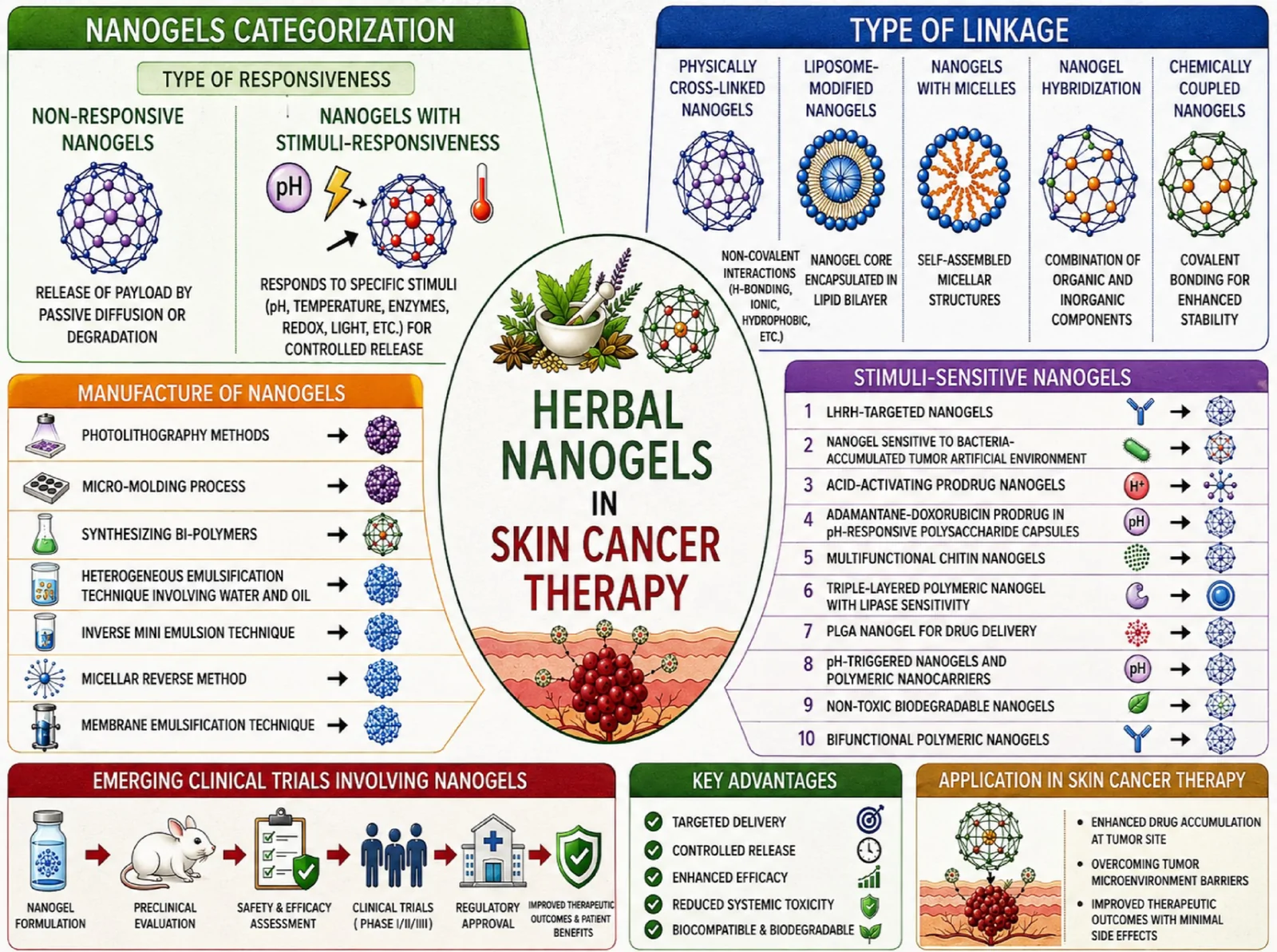

## Introduction

**Fig. 1:**
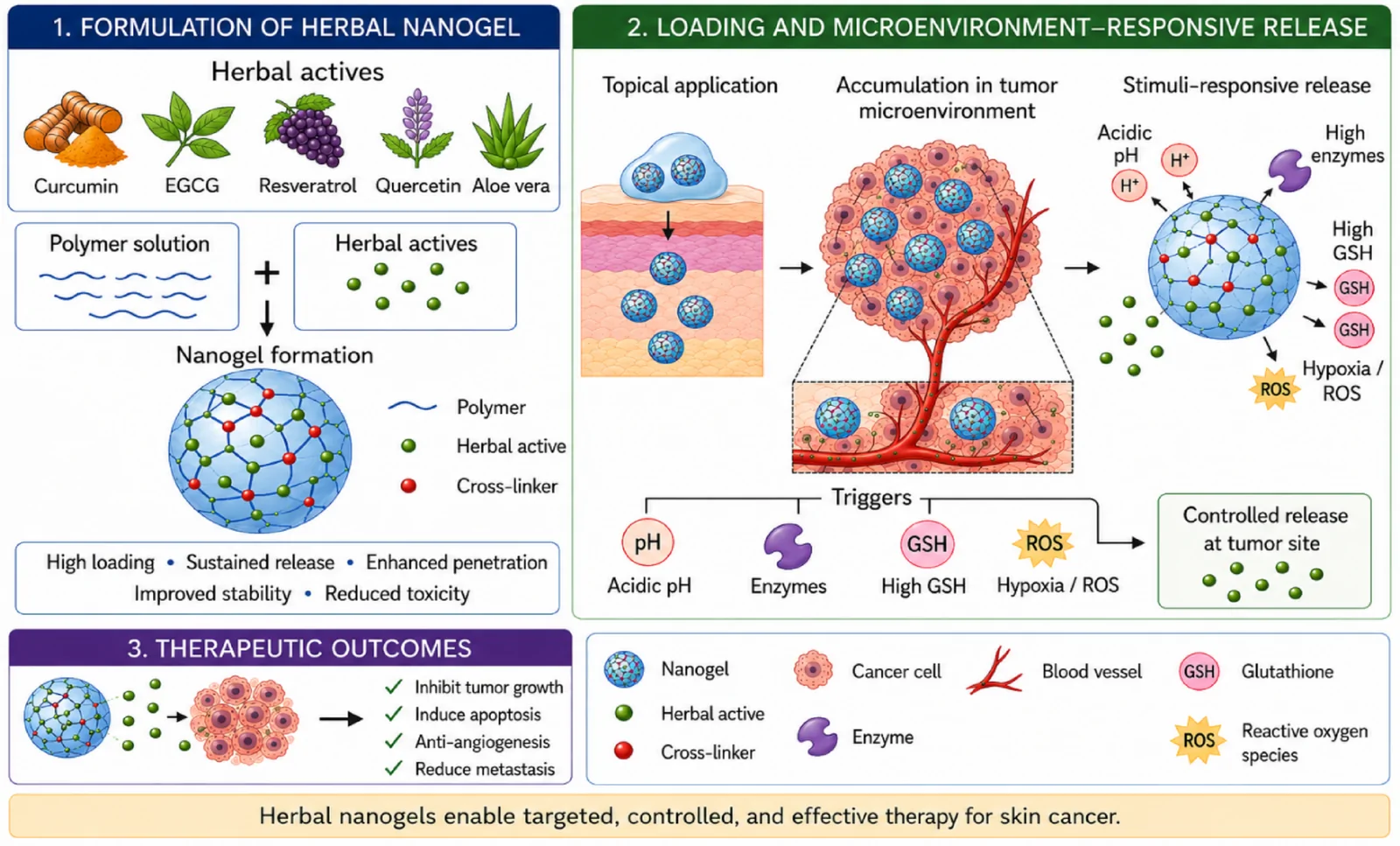
Schematic illustration of herbal nanogel-based skin cancer therapy showing (1) formulation of nanogels through the incorporation of herbal bioactives (curcumin, EGCG, resveratrol, quercetin, and aloe vera) into polymeric networks via cross-linking; (2) topical administration, tumor accumulation, and microenvironment-responsive release triggered by acidic pH, enzymes, elevated glutathione (GSH), and reactive oxygen species (ROS); and (3) therapeutic outcomes including inhibition of tumor growth, induction of apoptosis, suppression of angiogenesis, and reduction of metastasis. The nanogel platform enhances drug loading, stability, skin penetration, and controlled release while minimizing systemic toxicity

Healthcare professionals and researchers are exploring possibilities for innovative treatment techniques that might improve effectiveness while reducing undesirable side effects as a result of the worldwide growth in the prevalence of skin cancer, which offers a rising burden to the healthcare sector. In the past few decades, the convergence of nanotechnology and medicine has generated considerable interest because of its potential to provide novel solutions to longstanding therapeutic challenges [1]. Among these advances, herbal nanogel formulations have emerged as promising delivery systems for skin cancer therapy by integrating naturally derived bioactive compounds with nanotechnology-based carriers. Herbal constituents possessing antioxidant, anti-inflammatory, antiproliferative, and pro-apoptotic properties have attracted increasing attention for their potential to inhibit tumor progression while minimizing systemic toxicity [1]. Skin cancer is a group of malignancies arising from different cell types of the skin and includes melanoma and non-melanoma skin cancers [2]. Excision of the tumour remains the primary treatment approach; however, because of its invasive nature and the risk of deformity, alternative therapeutic strategies are needed [3]. Systemic chemotherapy remains effective but often leads to debilitating side effects owing to non-specific toxicity toward healthy tissues [3]. Radiation therapy is another widely used option, although its effectiveness is constrained by the risk of injury to surrounding healthy tissues. Consequently, research on nanotechnology-based approaches has accelerated, offering new opportunities for improving the management of skin cancer [4]. Particular attention has been directed toward herbal therapeutics such as curcumin, quercetin, resveratrol, epigallocatechin gallate, berberine, and aloe vera-derived phytoconstituents because of their demonstrated anticancer potential. Despite their therapeutic promise, many herbal compounds exhibit poor aqueous solubility, low bioavailability, and rapid degradation, which limit their clinical utility [5]. Encapsulation of these phytochemicals into nanogel systems represents an attractive strategy to overcome these limitations and enhance localized delivery to skin tumors [4]. Nanogels, which are nanosized cross-linked three-dimensional polymeric networks, are capable of encapsulating a wide variety of therapeutic agents and delivering them to the target site with high precision [4]. In herbal nanogel formulations, these networks serve as carriers for plant-derived bioactive molecules, providing sustained release, enhanced penetration through the skin barrier, improved stability, and reduced off-target toxicity. Such characteristics make herbal nanogels particularly suitable for topical and localized treatment of skin cancers [6]. Nanogels are responsive to various stimuli, including pH, temperature, and enzyme activity, thereby enabling controlled release of encapsulated agents. They are further classified into physically cross-linked gels, expandable liposomes containing nanogels, micelle-like nanogels, hybrid polymeric nanogels, and chemically cross-linked nanogels [6]. Various fabrication approaches, including photolithography, micro-molding, polymer preparation, emulsion-based techniques, and free-radical polymerization, are employed to tailor nanogel properties and optimize drug delivery performance [7]. Understanding these processes is essential for designing effective nanogel systems [8]. Recent developments have focused on the fabrication of herbal stimulus-responsive nanogels capable of releasing phytoconstituents selectively within the acidic and enzyme-rich tumor microenvironment. Such systems not only enhance therapeutic efficacy but also reduce undesirable systemic effects associated with conventional chemotherapy [9]. Furthermore, the incorporation of herbal molecules into pH-sensitive and enzyme-responsive nanogels has opened new avenues for achieving controlled and targeted treatment of skin malignancies [10]. Another important aspect of skin cancer treatment is the combination of herbal medicine with nanogel technology. The integration of traditional botanical knowledge with advances in nanotechnology has led to the development of multifunctional herbal nanogels capable of improving the therapeutic performance of phytochemicals. These systems have demonstrated significant potential in enhancing drug stability, prolonging retention at the tumor site, and increasing the effectiveness of natural anticancer agents [11]. Examples include curcumin-loaded nanogels, resveratrol-based nanogels, and quercetin-containing nanogel formulations designed for topical management of melanoma and other skin cancers [10]. Nanogels have emerged as promising carriers for localized drug delivery in cancer therapy owing to their ability to provide sustained release, enhance drug retention at the target site, and potentially reduce systemic exposure compared with conventional chemotherapy [12]. These properties have demonstrated encouraging therapeutic outcomes in several preclinical studies. Conventional topical dressings and conventional wound-based treatment approaches used in skin cancer management exhibit several limitations that restrict their effectiveness [13]. Traditional dressings primarily function as passive barriers and lack the ability to actively deliver therapeutic agents or respond to the tumor microenvironment. They often fail to provide controlled or sustained drug release, leading to suboptimal drug concentration at the target site [14]. In addition, poor penetration of active compounds through the stratum corneum further reduces therapeutic efficiency. Frequent dressing changes may also cause patient discomfort, increase the risk of infection, and delay wound healing. Moreover, conventional materials do not possess stimulus-responsive or targeted delivery capabilities, which limits their effectiveness in heterogeneous tumor tissues [15]. In many cases, they are unable to prevent tumor recurrence due to insufficient local drug retention and inadequate eradication of residual cancer cells. However, the purported advantages of localized nanogel delivery should be interpreted with caution, as their clinical benefits remain largely unvalidated. Concerns have been raised regarding formulation instability, batch-to-batch variability, limited penetration into heterogeneous tumor tissues, and the possibility that incomplete eradication of primary tumors may contribute to disease recurrence or facilitate metastatic progression under certain circumstances [16]. Furthermore, issues related to immunogenicity, long-term toxicity, and reproducibility continue to pose significant challenges to their successful translation into clinical practice. In contrast, systemic chemotherapy remains the cornerstone of treatment for advanced and metastatic cancers because of its ability to target disseminated tumor cells throughout the body [17]. Nevertheless, its therapeutic effectiveness is often constrained by dose-limiting toxicities and non-specific drug distribution. Consequently, recent research has increasingly focused on developing integrated therapeutic strategies that combine the localized and controlled delivery capabilities of nanogel-based systems with the broader anticancer activity of systemic therapies. Such combination approaches may offer improved therapeutic outcomes and reduced off-target effects; however, their long-term safety, pharmacokinetic behavior, and clinical efficacy require further investigation through rigorous preclinical and clinical studies [18]. These strategies include stimulus-responsive nanogels and the integration of localized therapy with immunotherapeutic approaches to elicit broader antitumor responses [13]. Given that extensive studies are still required to establish their safety and efficacy, these approaches remain highly attractive because of their potential to minimize systemic toxicity while maintaining therapeutic effectiveness [19]. The convergence of nanotechnology and herbal medicine has ushered in a new paradigm in skin cancer therapy. Herbal nanogels provide a versatile platform for the efficient delivery of phytochemicals, enabling enhanced bioavailability, targeted release, and improved therapeutic outcomes. Continued advancements in formulation strategies and a deeper understanding of the interactions between natural compounds and nanogel matrices are expected to accelerate the clinical translation of herbal nanogel systems [20]. Therefore, focuses specifically on recent advances in herbal nanogel formulations and highlights their emerging role in developing safe, effective, and minimally invasive therapeutic approaches for skin cancer management. Figure 1: Schematic representation of herbal nanogel-mediated skin cancer therapy. The illustration depicts (1) the fabrication of nanogels through encapsulation of herbal bioactive, including curcumin, epigallocatechin gallate (EGCG), resveratrol, quercetin, and aloe vera, within cross-linked polymeric networks; (2) topical delivery, accumulation within the tumor microenvironment, and stimuli-responsive drug release triggered by acidic pH, enzymatic activity, elevated glutathione (GSH) levels, and reactive oxygen species (ROS); and (3) therapeutic effects such as tumor growth inhibition, apoptosis induction, anti-angiogenic activity, and suppression of metastasis. Herbal nanogels improve drug loading capacity, physicochemical stability, skin penetration, and site-specific controlled release while reducing systemic toxicity. Table 1: Herbal Active Compounds Incorporated into Nanogels for Skin Cancer Therapy: Formulation Strategies and Therapeutic Outcomes.

**Table 1:** Nanogel-Based Delivery of Herbal Phytochemicals for Skin Cancer Therapy: Formulation Characteristics and Therapeutic Performance

Herbal Source	Active Molecule	Nanogel Type/Composition	Skin Cancer Model	Key In Vitro Outcomes	Key In Vivo Outcomes	Mechanism of Action	Reference
*Curcuma longa* (Turmeric)	Curcumin	Chitosan-based nanogel; thermo/pH-responsive nanogel	Melanoma (B16F10, A375 cells)	Enhanced cellular uptake, ROS generation, apoptosis induction, reduced viability	Significant tumor growth suppression in murine melanoma models	NF-κB inhibition, apoptosis induction, anti-inflammatory effects	[14]
*Vitis vinifera* (Grapes)	Resveratrol	Hyaluronic acid nanogel; polymeric nanogel	Melanoma and SCC	Increased cytotoxicity, inhibition of proliferation and migration	Reduced tumor burden and angiogenesis	Antioxidant activity, cell cycle arrest, anti-angiogenesis	[15]
*Berberis vulgaris*	Berberine	Chitosan/PVA nanogel	Melanoma cells	Enhanced apoptosis and mitochondrial dysfunction	Limited in vivo evidence available	AMPK activation, ROS-mediated apoptosis	[16]
*Camellia sinensis* (Green tea)	Epigallocatechin gallate (EGCG)	Gelatin-based nanogel	Melanoma models	Suppressed proliferation and oxidative stress	Improved topical retention and tumor inhibition	MAPK inhibition, antioxidant activity	[17]
*Aloe vera*	Aloin/Aloe-emodin	Hydrogel-nanogel hybrid system	Melanoma cells	Reduced cell viability and enhanced apoptosis	Preliminary animal studies show tumor regression	DNA intercalation, apoptosis induction	[18]
*Glycyrrhiza glabra* (Licorice)	Glycyrrhizin	Polymeric nanogel	Melanoma models	Anti-inflammatory and antiproliferative effects	Limited preclinical data	HMGB1 inhibition, immune modulation	[19]
*Scutellaria baicalensis*	Baicalein	Chitosan nanogel	B16 melanoma cells	Enhanced cellular uptake and apoptosis	Reduced tumor volume in mice	PI3K/Akt pathway inhibition	[20]
*Taxus brevifolia*	Paclitaxel (plant-derived)	Thermosensitive nanogel	Melanoma and SCC	Increased cytotoxicity and sustained release	Enhanced tumor regression and reduced systemic toxicity	Microtubule stabilization	[21]
*Withania somnifera* (Ashwagandha)	Withaferin A	Polymeric nanogel	Melanoma cells	Potent anticancer activity and apoptosis	Emerging preclinical evidence	ROS induction, inhibition of metastasis	[22]
*Azadirachta indica* (Neem)	Nimbolide	Nanostructured hydrogel/nanogel	Melanoma cells	Anti-proliferative and pro-apoptotic effects	Limited animal studies	STAT3 and NF-κB inhibition	[23]

## Nanogels categorization

**Fig. 2:**
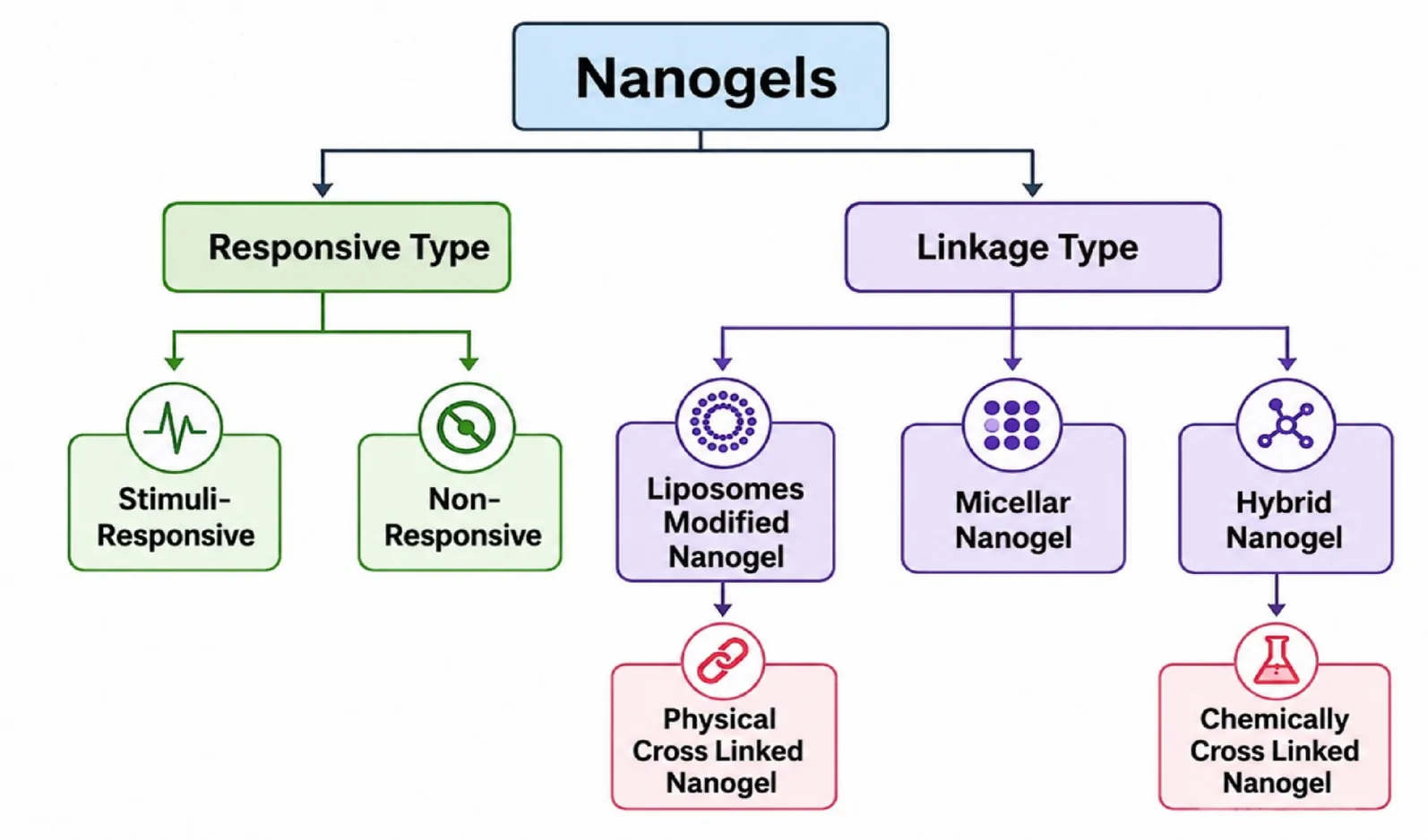
Categorisation of Nanogels by Responsiveness and Cross-linking

Based on their reactivity to external stimuli and the specific type of network-based bonding they have, nanogels may be roughly divided into two basic groups (Fig. 2) [14].

Through the use of this categorization, the individual might acquire an improved understanding of the structure-based, broad-spectrum, functional behaviour and applicability of these chemical compounds for certain pharmacological and biological applications. Table 2: Mechanistic Insights and Clinical Translation of Herbal Nanogel Formulations for Skin Cancer Management.

**Table 2:** Herbal Nanogel-Mediated Skin Cancer Therapy: Mechanistic Basis and Clinical Translation Prospects

Herbal Bioactive Compound	Nanogel Delivery System	Molecular Targets/Signaling Pathways	Mechanism of Anticancer Action	Tumor Microenvironment Interaction	Translational Significance	Ref
Curcumin	Chitosan, PLGA, PEGylated nanogels	MAPK/ERK, PI3K/Akt, NF-κB, STAT3	Induces apoptosis, inhibits cell proliferation and angiogenesis	ROS generation, acidic pH-triggered release	Enhanced bioavailability and sustained drug release	[14]
Quercetin	Chitosan and hyaluronic acid nanogels	PI3K/Akt, Bcl-2/Bax, Caspase-3	Cell cycle arrest and mitochondrial apoptosis	Redox-responsive intracellular release	Improved cellular uptake and reduced systemic toxicity	[15]
Resveratrol	PLGA and PEG nanogels	MAPK/ERK, NF-κB, SIRT1	Suppression of inflammation and tumor proliferation	Regulation of oxidative stress and cytokines	Increased stability and prolonged circulation	[16]
Berberine	pH-responsive polymeric nanogels	AMPK, PI3K/Akt/mTOR, NF-κB	Inhibition of metastasis and induction of apoptosis	pH-triggered release within tumor tissues	Targeted delivery and reduced adverse effects	[17]
Epigallocatechin gallate (EGCG)	Gelatin and dextran nanogels	MAPK, VEGF, NF-κB	Antiangiogenic and antiproliferative activity	Modulation of oxidative stress and inflammatory mediators	Improved solubility and therapeutic efficacy	[18]
Genistein	Chitosan nanogels	Akt, Caspase-9, Bax/Bcl-2	Activation of intrinsic apoptotic pathway	ROS-mediated cytotoxicity	Enhanced bioavailability and controlled release	[19]
Aloe vera phytoconstituents	Hydrogel and alginate nanogels	NF-κB, COX-2, TNF-α	Anti-inflammatory and antiproliferative effects	Modulation of inflammatory microenvironment	Improved local retention and skin penetration	[20]
Apigenin	Hyaluronic acid nanogels	MAPK/ERK, Caspase cascade	Induction of apoptosis and inhibition of melanoma growth	pH-sensitive release	Site-specific delivery and lower toxicity	[21]
Luteolin	Stimuli-responsive nanogels	PI3K/Akt, NF-κB, JAK/STAT	Inhibition of cell migration and proliferation	Suppression of ROS-mediated inflammation	Enhanced pharmacokinetic profile	[22]
Kaempferol	PEG-PLGA nanogels	ERK1/2, VEGF, MMP-9	Antiangiogenic and antimetastatic activity	Regulation of extracellular matrix remodeling	Increased therapeutic index	[23]

### Type of responsiveness

Nanogels can be categorized according to their ability to respond to external or internal stimuli, which is an important feature for achieving controlled and site-specific drug delivery. In herbal nanogel formulations, stimulus responsiveness plays a crucial role in regulating the release of phytoconstituents at the tumor site, thereby enhancing therapeutic efficacy and minimizing systemic toxicity.

#### Non-responsive nanogels

Non-responsive nanogels undergo passive swelling in the presence of water and other suitable solvents. Their behaviour is primarily governed by diffusion and absorption mechanisms and they do not respond to external stimuli [24]. These systems provide predictable and sustained drug release profiles, making them suitable for applications requiring prolonged therapeutic action [25]. However, the lack of responsiveness limits their utility in targeted therapies, particularly in complex pathological conditions such as cancer. Non-responsive nanogels have been investigated as carriers for plant-derived bioactive compounds to achieve sustained topical delivery. Such systems may improve the stability and retention of phytochemicals on the skin surface; however, their inability to respond to tumor-specific conditions restricts their effectiveness compared with smart nanogel systems.

#### Stimuli-responsive nanogels

Stimuli-responsive nanogels undergo reversible physicochemical changes, such as swelling or shrinkage, in response to environmental triggers including pH, temperature, ionic strength, magnetic fields, electromagnetic radiation, and enzymatic activity [26]. These smart nanogels possess enormous potential for cancer therapy because they can release therapeutic agents selectively at the tumor site in response to cancer-associated conditions such as acidic pH and elevated temperature [27]. Multifunctional polymeric nanogels capable of responding to multiple stimuli further enhance targeting accuracy and therapeutic efficacy [28]. Their applications include controlled drug delivery, imaging, and theragnostic systems. In herbal nanogel formulations, stimulus-responsive systems are particularly advantageous for improving the delivery of phytochemicals such as curcumin, quercetin, resveratrol, berberine, epigallocatechin gallate, and other natural anticancer agents. The acidic and enzyme-rich tumor microenvironment of skin cancer can trigger the controlled release of encapsulated herbal constituents, thereby increase their local concentration and therapeutic effectiveness while reduce off-target toxicity [29]. Consequently, pH-responsive and enzyme-sensitive herbal nanogels have emerged as promising platforms for melanoma and non-melanoma skin cancer therapy.

### Type of linkage

Nanogels are also classified according to the nature of the cross-linking interactions that form the three-dimensional polymeric network. These interactions influence the mechanical properties, stability, swelling behaviour, and drug-release characteristics of nanogels. Herbal nanogel formulations, the type of linkage significantly affects the encapsulation efficiency, stability, and controlled release of phytoconstituents intended for skin cancer therapy.

#### Physically cross-linked nanogels

Physically cross-linked nanogels, commonly referred to as pseudogels, are formed through weak non-covalent interactions such as hydrophobic interactions, electrostatic attractions, hydrogen bonding, and van der Waals forces [30]. Their preparation is relatively simple and does not require chemical cross-linking agents [31], making them environmentally friendly and less toxic. Their properties can be modulated by factors including polymer concentration, pH, temperature, and ionic strength [32]. These nanogels are commonly formed by self-assembly of amphiphilic block copolymers or by electrostatic complexation between oppositely charged polymers [33]. However, compared with chemically cross-linked systems, they generally exhibit lower mechanical strength and stability [34]. Physically cross-linked herbal nanogels have attracted considerable interest owing to their biocompatibility and mild fabrication conditions, which preserve the biological activity of sensitive phytochemicals. Natural polymers such as chitosan, alginate, gelatin, and hyaluronic acid have been extensively explored for encapsulating herbal compounds with anticancer potential [35]. These systems provide sustained release and improved skin permeation, making them attractive for topical skin cancer therapy.

#### Liposome-modified nanogels

Liposome-modified nanogels have demonstrated considerable promise as drug carriers for the treatment of various diseases. The release of liposomes embedded within the nanogel matrix is regulated by several factors, including pH, temperature, and ionic charge. For example, succinylated poly(glycerol)-containing liposomes have been shown to enhance cytoplasmic delivery of calcein at pH 5.5 [36]. Poly(N-isopropylacrylamide)-based liposome-modified nanogels also exhibit thermoresponsive drug-release behaviour following subcutaneous administration [37]. The incorporation of herbal compounds into liposome-modified nanogels offers additional advantages by combining the high loading capacity of nanogels with the membrane-mimicking characteristics of liposomes [38]. Such hybrid systems can improve the solubility, stability, and cellular uptake of hydrophobic phytoconstituents, thereby enhancing their therapeutic potential against skin cancer [38].

#### Micellar nanogels

**Fig. 3:**
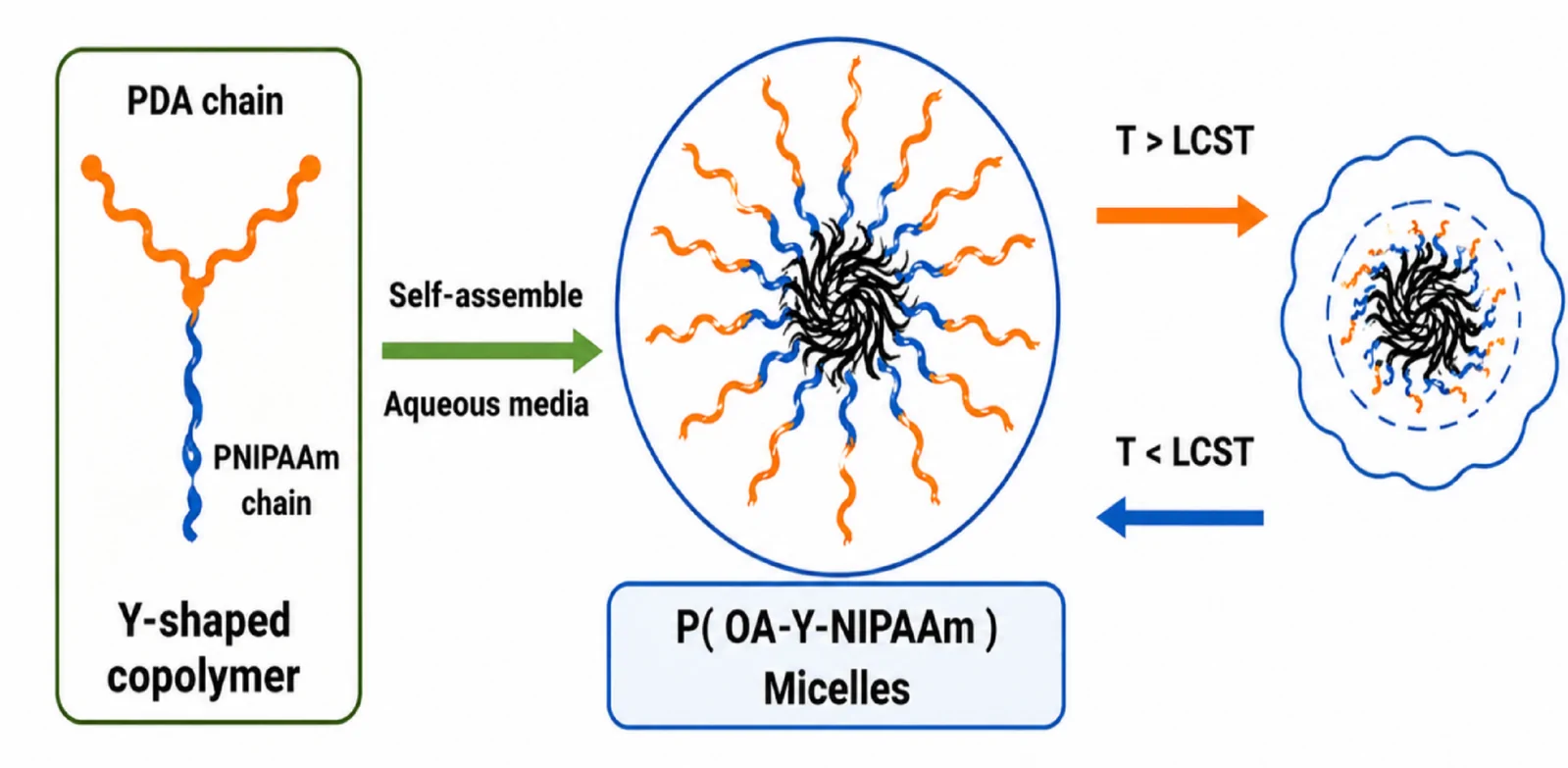
Schematic of the Formation of Thermo-Responsive Micellar Nanogel

Micellar nanogels are formed through the self-assembly of amphiphilic block copolymers or graft copolymers in aqueous media. These nanogels exhibit a characteristic core-shell structure in which the hydrophobic core is surrounded by a hydrophilic polymer shell [39]. The hydrophilic component facilitates hydrogen bonding and contributes to the stability of the micellar structure. Core-shell micellar nanogels possess excellent stability and responsiveness to external stimuli [40]. Their large surface area enables efficient loading of therapeutic agents through physical entrapment, while the hydrophobic core protects encapsulated compounds from enzymatic degradation and hydrolysis [41]. Researchers are investigating Y-shaped nanoparticles composed of biodegradable and biocompatible polymers such as PLGA, chitosan, and oleic acid-isopropylacrylamide copolymers for efficient drug delivery (Fig. 3) [42]. Micellar herbal nanogels are particularly suitable for the encapsulation of poorly water-soluble phytochemicals commonly used in skin cancer therapy. Bioactive compounds such as curcumin, resveratrol, quercetin, and berberine can be effectively incorporated within the hydrophobic core, resulting in enhanced stability, improved bioavailability, and prolonged release profiles [43]. These characteristics contribute to increased accumulation of herbal agents at tumor sites and represent a promising strategy for the treatment of melanoma and other skin malignancies.

#### Nanogel hybridization

**Fig. 4:**
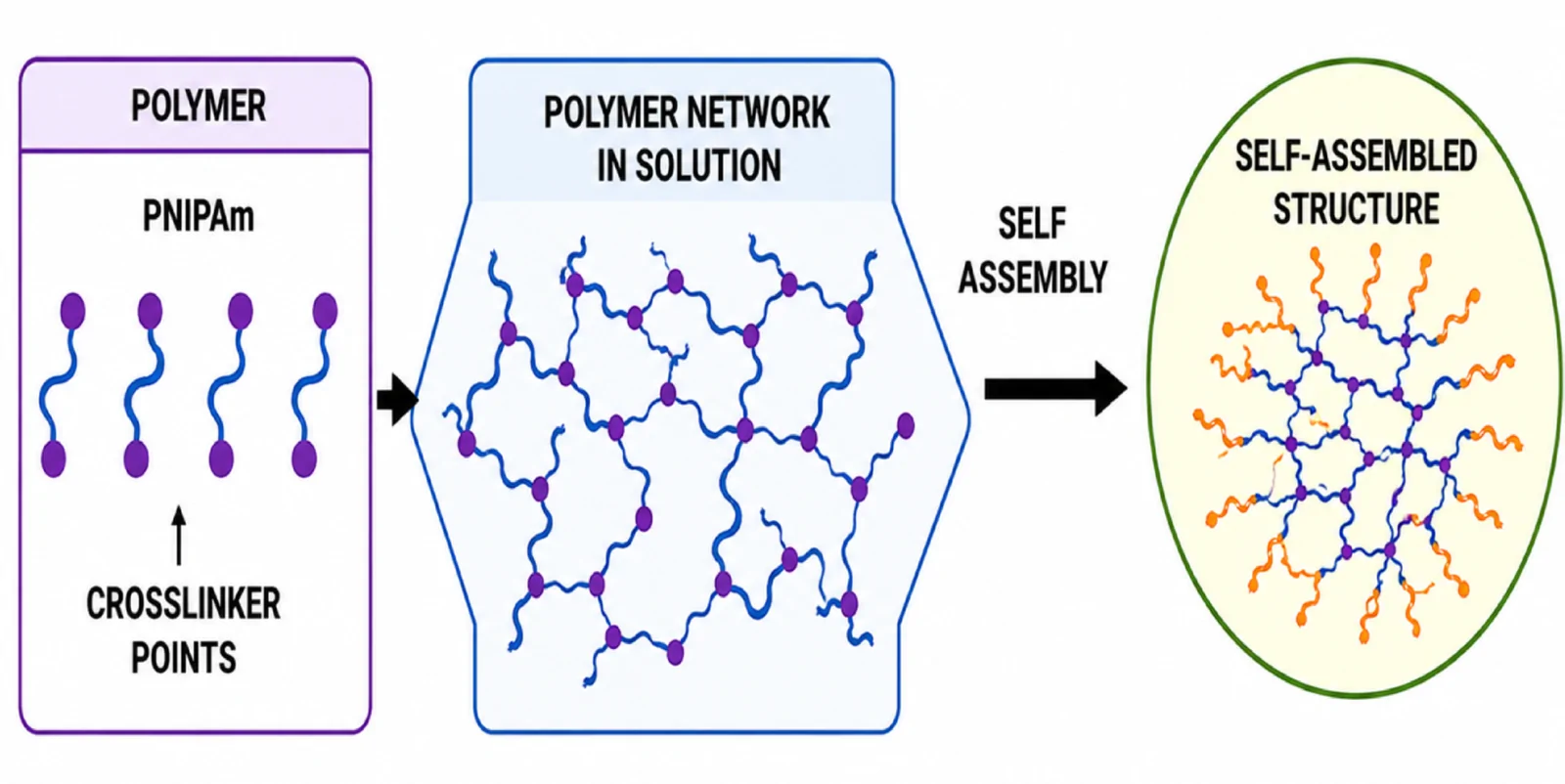
Illustration of Self-Assembly of Polymers into Nanostructured Systems

Hybrid nanogels are fabricated by incorporating nanoparticles into organic or inorganic polymeric matrices, thereby integrating the complementary properties of multiple materials within a single multifunctional platform. These hybrid systems exhibit high sensitivity to environmental cues and can respond to a variety of physiological stimuli. Hybrid nanogels formed through the self-assembly of polymers in aqueous media have demonstrated remarkable efficiency in the rapid and targeted delivery of therapeutic agents, as illustrated in Fig. 4 [44]. Various polymers, including polysaccharides, pullulan-PNIPAM, and related materials, have been employed in the fabrication of hybrid nanogels [45]. Among these systems, cholesterol-pullulan hybrid nanogels have been extensively investigated and have shown significant potential as drug delivery vehicles for genetic materials, RNA, proteins, peptides, oligosaccharides, and other bioactive compounds [46]. Furthermore, hybrid nanogels have demonstrated the capability for site-specific and stimuli-responsive delivery of insulin and anticancer agents. Numerous studies have reported that cholesterol-pullulan hybrid nanogels can effectively encapsulate and transport genetic materials, RNA, peptides, fatty acids, and other therapeutic molecules. These nanogel systems have also been explored for the localized and controlled delivery of anticancer drugs, highlighting their potential in precision medicine and targeted therapy [47] Hybrid nanogels represent highly versatile carriers for the encapsulation and delivery of phytoconstituents used in skin cancer therapy. The combination of polymeric matrices with inorganic nanoparticles or lipid-based components enhances the stability, loading capacity, and controlled release characteristics of natural bioactive compounds [48]. Herbal molecules such as curcumin, quercetin, resveratrol, epigallocatechin gallate, and berberine can be efficiently incorporated into hybrid nanogel systems to overcome limitations associated with poor solubility, low bioavailability, and rapid degradation [49]. In addition, hybrid herbal nanogels facilitate enhanced skin penetration and prolonged retention of phytochemicals at the tumor site, thereby improving therapeutic efficacy while minimizing systemic toxicity [50]. The integration of natural polymers, such as chitosan and polysaccharides, with synthetic polymers or inorganic nanomaterials further provides multifunctional properties, including stimulus-responsive release and improved biocompatibility [51]. Consequently, hybrid herbal nanogels have emerged as promising platforms for the localized treatment of melanoma and non-melanoma skin cancers and represent an important area of ongoing research in nanomedicine [51].

#### Chemically coupled nanogels

**Fig. 5:**
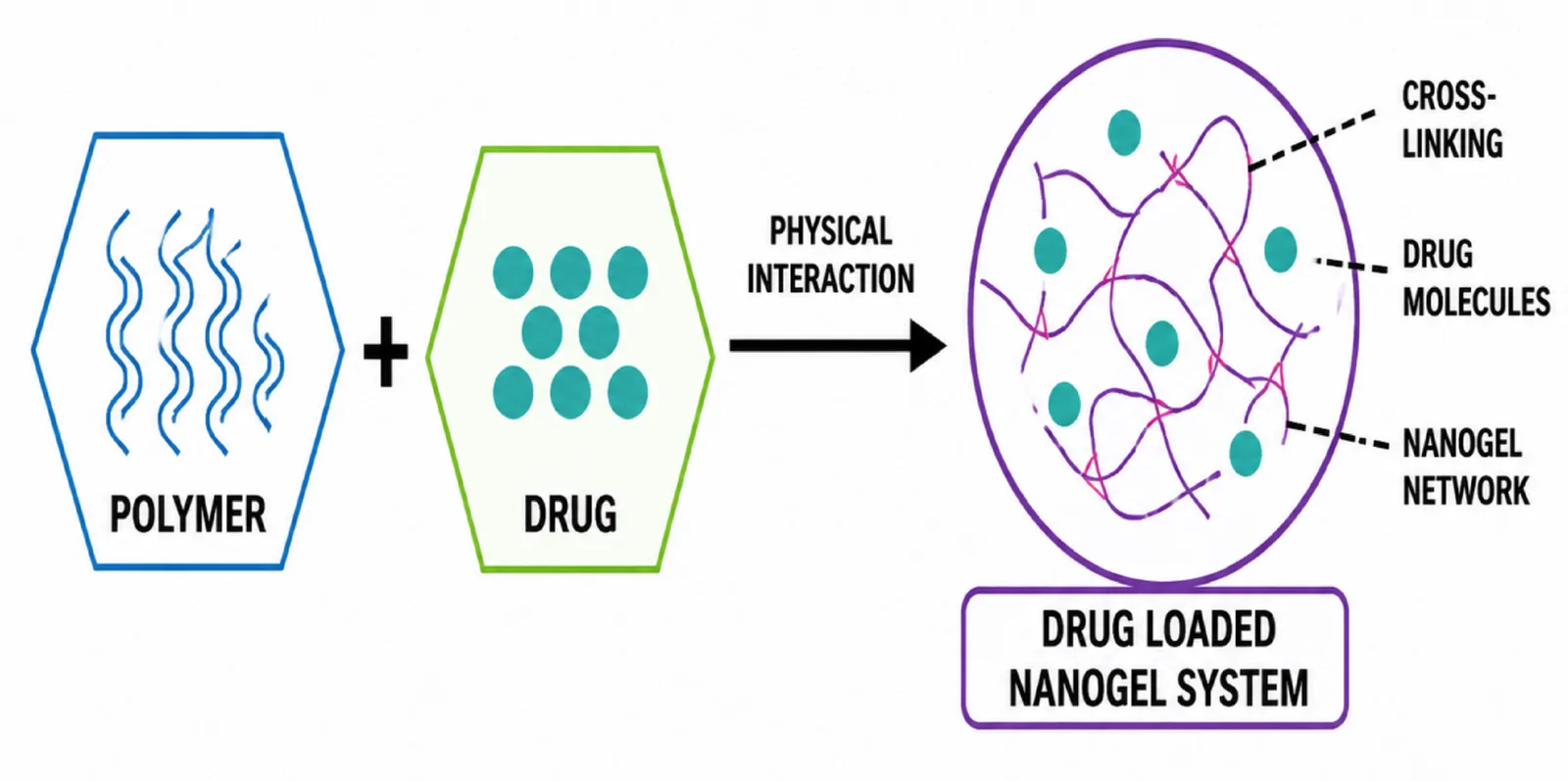
Schematic illustration of drug-loaded nanogel formation showing the interaction between polymer chains and drug molecules through chemical transformation that leads to a cross-linked nanogel network and drug molecules trapped within a three-dimensional network

Chemically cross-linked nanogels are synthesized through covalent bonding between polymer chains. The morphology and properties of these gels depend on the nature of the functional groups present in the polymeric matrix and the strength of the chemical interactions involved (Fig. 5) [52]. This approach has proven effective for the fabrication of a wide range of nanogel systems. The incorporation of multifunctional cross-linking agents facilitates the formation of hydrophilic and amphiphilic copolymeric networks when suitable vinyl monomers are employed [53]. The physicochemical characteristics and stability of nanogel formulations are largely dependent on the type of cross-linkers used [54]. Furthermore, the dimensions, architecture, and morphological properties of nanogels are significantly influenced by the cross-linking mediators. Sustainable PLGA-PEG diblock nanogels containing expandable pendant thiol groups have been successfully prepared using disulfide cross-linking strategies [55]. These nanogels were designed with particle sizes ranging from 20 to 200 nm. In addition, environmentally friendly polymerization approaches based on green chemistry principles have been employed during their synthesis [56]. Chemically cross-linked nanogels offer superior structural integrity, controlled drug release, and enhanced stability compared with physically cross-linked systems. These characteristics are particularly advantageous for the encapsulation of plant-derived bioactive compounds used in skin cancer therapy. Phytochemicals such as curcumin, quercetin, resveratrol, berberine, epigallocatechin gallate, and other naturally occurring anticancer agents often suffer from poor aqueous solubility and rapid degradation, which limit their therapeutic efficacy [57]. Chemically coupled nanogels provide a stable three-dimensional network capable of protecting these phytoconstituents and ensuring their sustained and site-specific release [58]. Moreover, the use of biodegradable polymers such as PLGA, PEG, chitosan, and hyaluronic acid enable the development of biocompatible herbal nanogel systems with improved skin penetration and prolonged residence time at the tumor site [59]. Stimuli-responsive chemically cross-linked nanogels can further exploit the acidic and enzyme-rich microenvironment of skin tumors to achieve selective release of encapsulated phytochemicals. Such properties contribute to enhanced anticancer activity, reduced systemic toxicity, and improved patient compliance [60]. Consequently, chemically coupled herbal nanogels have emerged as promising platforms for the localized treatment of melanoma and non-melanoma skin cancers and represent an important area of investigation in contemporary nanomedicine [39].

## The manufacture of nanogels

The fabrication process plays a critical role in determining the physicochemical characteristics, drug-loading capacity, stability, and release behaviour of nanogel formulations. In herbal nanogel-based skin cancer therapy, the selection of an appropriate manufacturing technique is particularly important for preserving the biological activity of phytoconstituents and ensuring efficient delivery to tumor tissues. Various fabrication approaches have been employed to produce herbal nanogels with improved therapeutic performance and controlled drug-release properties.

### Photolithography method

Photolithography is one of the techniques employed for the fabrication of three-dimensional nanogel structures. Photolithographically prepared polymeric molds possess a large surface area, enabling the incorporation and retention of substantial amounts of therapeutic agents. The photolithographic process consists of five major steps.

1. A UV-cross-linkable polymer, acting as a substrate with a high surface area, is immersed in water for a prolonged period and coated with a previously dried photosensitive material [40]. Subsequently, a quartz template is carefully pressed onto the polymer to generate a wafer-like structure [41].
2. The molded template is then exposed to ultraviolet radiation.
3. After exposure, the quartz pattern is separated from the sample while maintaining a thin intervening film layer [42].
4. In the next stage, the residual thin film is removed using oxygen plasma treatment.
5. Finally, the substrate is dissolved in aqueous buffer systems under controlled conditions to recover the synthesized particles [43].

Photolithography offers several advantages over conventional printing techniques because it enables the fabrication of particles with well-defined size and geometry without requiring template wetting [44]. The Particle Replication in Non-Wetting Templates (PRINT) technology makes it possible to fabricate nanogel systems with controlled dimensions, morphology, and stability. This technique has demonstrated considerable potential for the precise delivery of therapeutic molecules, including drugs, fatty acids, genetic material, proteins, and RNA [45]. Photolithographic approaches provide an efficient platform for producing nanogels with uniform particle size, high loading efficiency, and reproducible release profiles. These characteristics are particularly beneficial for encapsulating plant-derived bioactive compounds used in skin cancer therapy [61]. Phytochemicals such as curcumin, quercetin, resveratrol, berberine, and epigallocatechin gallate are susceptible to degradation and exhibit poor aqueous solubility. Therefore, the ability of photolithographic techniques to generate structurally uniform nanogels contributes to enhanced stability, improved bioavailability, and controlled release of these herbal constituents [61]. Furthermore, the precise control over particle architecture offered by PRINT technology facilitates targeted and localized delivery of phytochemicals to melanoma and non-melanoma skin cancer tissues. Such herbal nanogel systems can enhance skin penetration, prolong residence time at the tumor site, and minimize systemic toxicity [61]. Consequently, photolithography-based fabrication methods represent promising strategies for the development of advanced herbal nanogel formulations aimed at improving the therapeutic outcomes of skin cancer treatment [45].

### Micro-molding process

Although photolithography and micro-molding share certain similarities, micro-molding offers advantages in terms of cost-effectiveness and ease of fabrication [46]. A variety of particle geometries, including hexagonal prisms, discs, and string-like structures, can be generated using micro-molding techniques. During the fabrication process, suspended cells are dispersed within aqueous precursor solutions containing photo-initiators. These precursor systems commonly include PEGDA (polyethylene glycol diacrylate), methaacrylated hyaluronic acid (MeHA), and related compounds [47]. Subsequently, ultraviolet light-induced photocrosslinking is employed to form hydrogel structures within plasma-treated hydrophilic PDMS molds. The resulting microgel particles containing encapsulated cellular components are then isolated, washed, and collected [48]. Micro-molding represents a versatile approach for producing particles with precisely controlled shapes, sizes, and drug-loading characteristics. Such control is particularly important for preserving the stability and biological activity of plant-derived bioactive compounds used in skin cancer therapy. The technique allows the fabrication of uniform nanogel structures capable of efficiently encapsulating phytochemicals, thereby improving their therapeutic performance [49]. Furthermore, micro-molding-based herbal nanogels can enhance the solubility, stability, and sustained release of natural anticancer agents such as curcumin, quercetin, resveratrol, berberine, and epigallocatechin gallate [50]. The ability to tailor particle morphology and size distribution facilitates improved skin penetration and prolonged retention of phytoconstituents at tumor sites. These features are particularly advantageous for the localized treatment of melanoma and non-melanoma skin cancers, where sustained exposure to herbal therapeutics can enhance antitumor efficacy while minimizing systemic toxicity [51]. In addition, the biocompatibility of polymers such as PEGDA and methaacrylated hyaluronic acid makes micro-molding an attractive strategy for developing multifunctional herbal nanogels with potential applications in targeted drug delivery and skin cancer therapy [48]. Consequently, micro-molding techniques provide an effective platform for the fabrication of advanced herbal nanogel systems with enhanced therapeutic potential [48].

#### Method for synthesizing bi-polymers

Natural biopolymers derived from carbohydrates play an important role in the fabrication of nanogel systems. Commonly employed biopolymers include chitosan, albumin, hyaluronic acid, and dextran. Owing to their excellent biocompatibility, biodegradability, and low toxicity, these materials have been extensively utilized for the preparation of nanogels, microgels, and hydrogels [61]. Various fabrication approaches, including self-assembly and swelling techniques, have been employed to successfully produce these systems from natural biopolymers [4]. The use of natural biopolymers is particularly advantageous in the development of herbal nanogel formulations for skin cancer therapy. These materials provide a suitable matrix for the encapsulation of plant-derived bioactive compounds and contribute to improved drug stability, enhanced skin permeability, and sustained release characteristics [62]. Moreover, the inherent biocompatibility of natural polymers minimizes adverse effects and supports their application in topical and localized cancer treatment. Several approaches have been developed for the preparation of nanogels based on natural polymers, which may be broadly classified as follows (1) Spray-drying technique [23] (2) Homogeneous aqueous gelation [49] (3) Chemical cross-linking of dextran [24]. Among these approaches, polysaccharide-based systems such as chitosan-, dextran, and hyaluronic acid-derived nanogels have attracted considerable attention for delivering phytochemicals with anticancer activity [63]. Natural compounds including curcumin, quercetin, resveratrol, berberine, epigallocatechin gallate, and aloe vera constituents can be effectively incorporated into these biopolymeric matrices to overcome limitations associated with poor aqueous solubility, rapid degradation, and limited bioavailability [64]. Furthermore, natural biopolymer-based herbal nanogels exhibit excellent mucoadhesive and skin-adhesive properties, facilitating prolonged retention of therapeutic agents at the tumor site. Their ability to provide controlled release and enhanced penetration through the skin barrier makes them promising carriers for the treatment of melanoma and non-melanoma skin cancers [65]. Consequently, natural biopolymer-derived nanogels represent an important class of delivery systems for improving the therapeutic efficacy of herbal medicines in skin cancer management [66].

### Heterogeneous emulsification technique involving water and oil

The water-in-oil (W/O) heterogeneous emulsification method is one of the most widely employed approaches for the fabrication of nanogel systems. In this technique, aqueous solutions containing hydrophilic biopolymers are dispersed within a continuous lipophilic phase using suitable lipid-soluble surfactants [67]. Subsequently, cross-linking of the biopolymeric network is achieved through the use of hydrophilic cross-linking agents, leading to the formation of stable nanogel particles [68]. The W/O heterogeneous emulsification technique has gained considerable importance in the preparation of herbal nanogel formulations because it enables efficient encapsulation of plant-derived bioactive compounds while preserving their physicochemical stability [69]. The method offers excellent control over particle size and morphology, thereby facilitating the production of nanogels with enhanced drug-loading capacity and sustained-release characteristics [69]. Natural polymers such as chitosan, dextran, albumin, and hyaluronic acid are frequently employed in this method to fabricate biocompatible herbal nanogels [70]. These systems can effectively incorporate phytochemicals with anticancer potential, including curcumin, quercetin, resveratrol, berberine, and epigallocatechin gallate, thereby overcoming challenges associated with poor aqueous solubility, instability, and limited bioavailability [71]. Furthermore, herbal nanogels prepared through W/O emulsification exhibit improved skin penetration and prolonged retention of phytoconstituents at the tumor site, which are desirable characteristics for the localized treatment of melanoma and non-melanoma skin cancers [72]. The controlled release properties of these formulations contribute to enhanced therapeutic efficacy while minimizing systemic toxicity.

### Inverse mini emulsion technique

Inverse miniemulsion techniques involve the dispersion of aqueous biopolymeric solutions within a continuous hydrophobic phase using high-speed homogenizers, agitators, or continuous stirring processes. Water-insoluble surfactants, such as Span-80 and Aerosol OT, are employed to stabilize the resulting microemulsion system, thereby facilitating the formation of nanogel matrices [51]. These microemulsions enable the incorporation of various biologically active molecules, including RNA, drugs, and genetic materials, into hydrophilic particles [52]. Subsequently, chemical cross-linking agents such as glutaraldehyde are utilized to stabilize the hydrophilic particles. The resulting microgel particles are subjected to precipitation, centrifugation, and repeated washing with appropriate organic solvents, including isopropanol, before undergoing lyophilization [53]. Miniemulsion technology has also been widely employed in the preparation of gelatin-based nanogel systems [54]. The process primarily involves fusion and fission mechanisms. Persistent nanogel structures can be obtained by fusing miniemulsions of polymeric solutions, such as gelatin and poly(butylene-co-ethylene)-block-poly (ethylene oxide), followed by ultrasonic treatment and cross-linking with glutaraldehyde [55]. These gel particles generally exhibit particle sizes ranging from 100 to 400 nm. As demonstrated by Kim and co-workers, a relatively high concentration of surfactants is required in miniemulsion systems to obtain stable gel particles [56]. This technique allows the preparation of particles within both the nanometer and micrometre ranges. Inverse miniemulsion techniques provide an efficient platform for encapsulating phytoconstituents with poor aqueous solubility and limited bioavailability. The method offers excellent control over particle size distribution and enables the production of stable nanogel systems capable of protecting sensitive herbal molecules from degradation [57]. Natural bioactive compounds such as curcumin, quercetin, resveratrol, berberine, epigallocatechin gallate, and aloe vera-derived constituents can be successfully incorporated into miniemulsion-based nanogels. Encapsulation within these polymeric matrices improves the stability, skin permeation, and sustained-release behaviour of phytochemicals, thereby enhancing their therapeutic potential against melanoma and non-melanoma skin cancers [58]. Furthermore, gelatin-based and polysaccharide-derived herbal nanogels prepared by inverse miniemulsion techniques exhibit excellent biocompatibility and biodegradability, making them attractive carriers for localized drug delivery [59. The nanoscale dimensions achieved through this approach facilitate enhanced penetration into skin tissues and prolonged retention at tumor sites, thereby increasing anticancer efficacy while minimizing systemic adverse effects. Consequently, inverse miniemulsion technology represents a promising strategy for the development of advanced herbal nanogel formulations for skin cancer therapy [60].

### Micellar reverse method

**Fig. 6:**
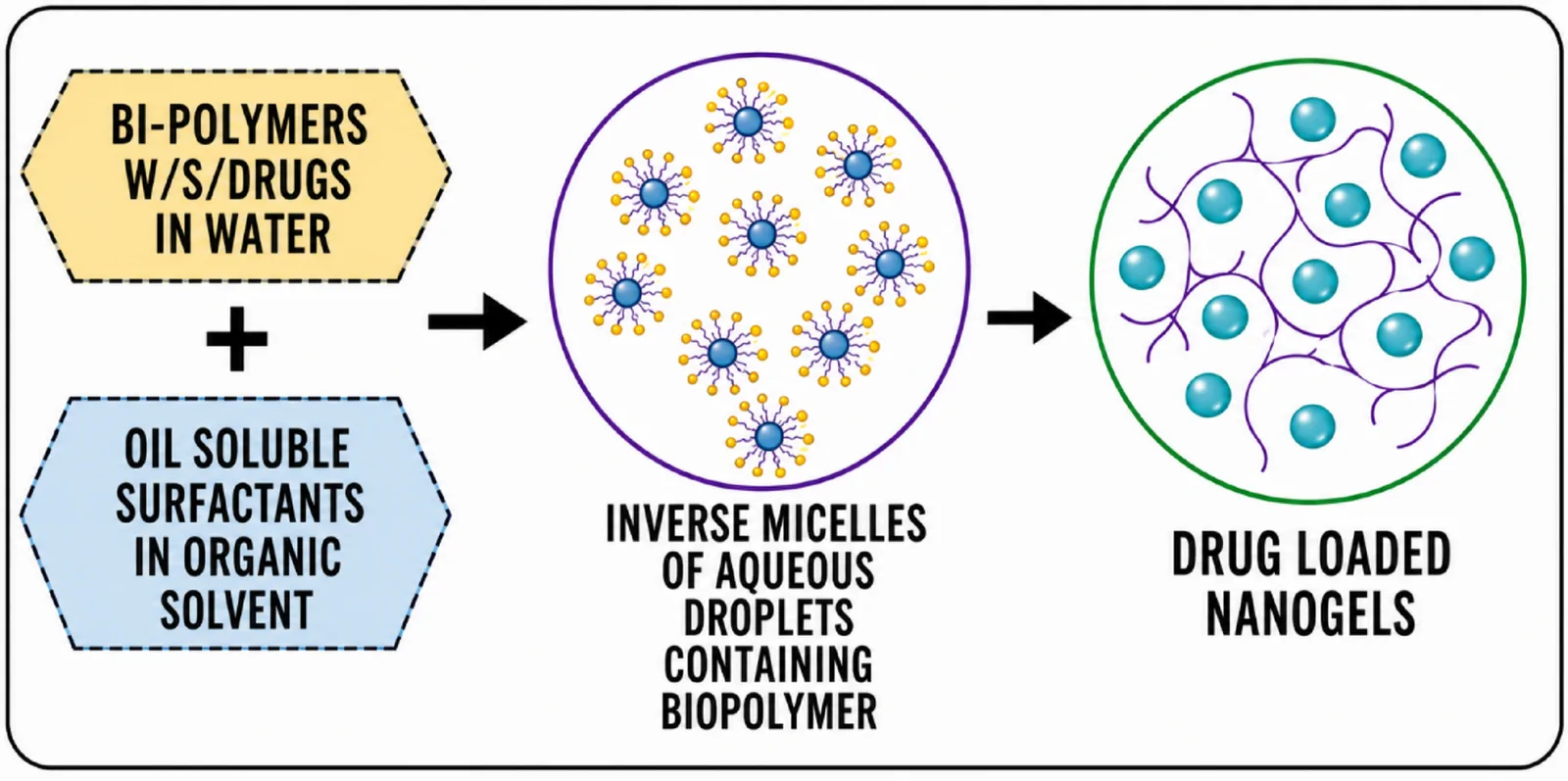
Schematic representation of preparation of drug-loaded nanogels using the inverse micelle technique, which involves mixing of biopolymers with drug in water and oil-soluble surfactants in organic solvent to form inverse micelles containing biopolymer, and eventually resulting in drug-loaded nanogels

The reverse micellar technique is based on the water-in-oil (W/O) dispersion phenomenon and is similar to the inverse miniemulsion approach. In this method, a large amount of lipophilic surfactant is employed to produce a thermodynamically stable micellar system consisting of water-containing droplets dispersed throughout a continuous oil phase [57]. The resulting gel particles are highly compact and generally exhibit particle sizes ranging from 10 to 150 nm (Fig. 6). The formation of uniformly sized particles requires suitable cross-linking agents, while an appropriate concentration of oil-soluble surfactants is essential for obtaining thermodynamically stable nanosized systems [58]. The reverse micellar technique has attracted considerable attention in the fabrication of herbal nanogels because it enables the preparation of particles with narrow size distribution and high encapsulation efficiency [61]. Such characteristics are particularly advantageous for incorporating phytochemicals intended for skin cancer therapy, as many natural compounds suffer from poor aqueous solubility and limited bioavailability. Herbal bioactive compounds, including curcumin, quercetin, resveratrol, berberine, epigallocatechin gallate, and aloe vera-derived constituents, can be effectively entrapped within reverse micelle-based nanogel systems [62]. Encapsulation within these nanosized carriers enhances the physicochemical stability of phytochemicals, protects them against premature degradation, and facilitates sustained and controlled release. Furthermore, the small particle size obtained through the reverse micellar approach promotes enhanced penetration through the skin barrier and improves the accumulation of phytoconstituents at tumor sites [63]. These properties are particularly beneficial for the treatment of melanoma and non-melanoma skin cancers, where localized delivery and prolonged retention of therapeutic agents are desirable. Natural polymers such as chitosan, dextran, hyaluronic acid, and gelatin are frequently utilized in reverse micellar systems owing to their excellent biocompatibility and biodegradability [64]. The combination of these polymers with herbal bioactive compounds results in multifunctional nanogel systems capable of improving therapeutic efficacy while reducing systemic toxicity. Consequently, reverse micellar techniques represent a promising strategy for the development of advanced herbal nanogel formulations for targeted skin cancer therapy [64].

### Membrane emulsification technique

**Fig. 7:**
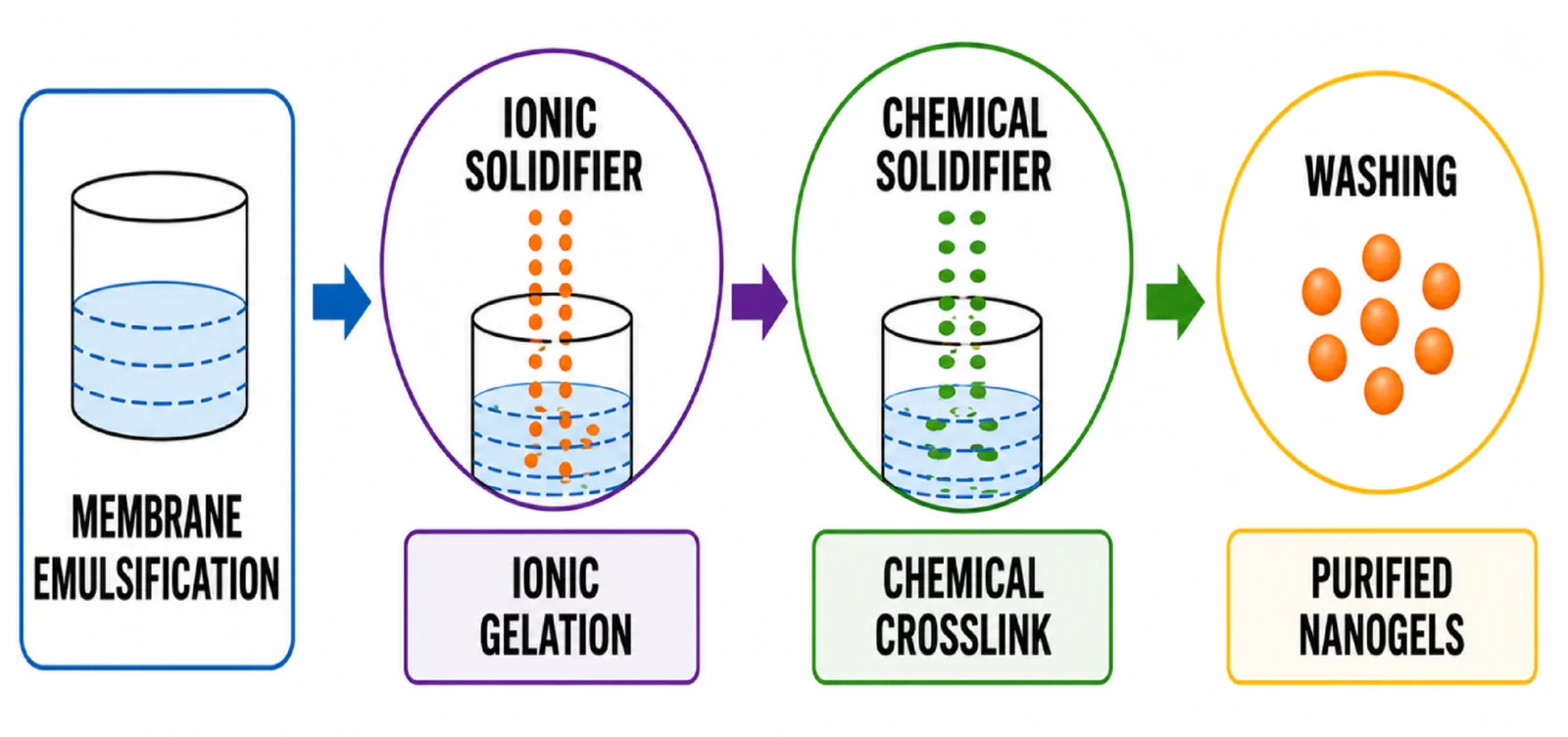
Fabrication of nanogels using membrane emulsification and two-step solidification processes. The scheme depicts the processes of ionic gelation with an ionic solidifier, followed by chemical crosslinking with a chemical solidifier, and finally washing to yield purified nanogels

The membrane emulsification technique is an efficient approach for producing nanogel particles with narrow size distributions and highly dispersed spherical morphologies. This method utilizes a membrane filtration process involving Shirasu porous glass (SPG) membranes containing uniformly distributed pores ranging from 0.1 to 20 μm [59]. Under controlled pressure conditions, droplets of similar sizes are transported through the membrane to form either water-in-oil (W/O) or oil-in-water (O/W) emulsions with uniform particle size distributions (Fig. 7). Furthermore, this technique can be employed for the preparation of various emulsion systems, including solid/O/W, O/W/O, and W/O/W dispersions [60]. The resulting particles can exhibit diverse morphologies, including microspheres, hollow particles, crystalline structures, and core-shell microparticles [61]. Subsequently, thermally stable nanogels are produced by combining membrane emulsification with sequential cross-linking strategies [62]. Initially, ions undergo gelation to generate fine nanoparticles, which are dispersed within ionic hardening solutions and then subjected to chemical cross-linking. Finally, the particles are purified and collected [63]. Membrane emulsification offers several advantages, including precise control over particle size, enhanced encapsulation efficiency, and improved reproducibility. These characteristics are particularly important for preserving the biological activity of plant-derived phytochemicals intended for skin cancer therapy [64]. The technique is highly suitable for incorporating natural anticancer compounds such as curcumin, quercetin, resveratrol, berberine, epigallocatechin gallate, and aloe vera-derived constituents into nanogel matrices. Encapsulation within membrane-emulsified nanogels enhances the stability and solubility of these phytochemicals while protecting them from premature degradation [65]. Moreover, the formation of homogeneous particles facilitates controlled and sustained drug release, leading to prolonged retention of herbal compounds at the tumor site. Such properties are advantageous for topical and localized treatment of melanoma and non-melanoma skin cancers, where enhanced skin penetration and site-specific delivery are essential for maximizing therapeutic efficacy and minimizing systemic adverse effects [66]. Natural polymers such as chitosan, hyaluronic acid, dextran, and gelatin are frequently employed in membrane-emulsification-based herbal nanogels owing to their excellent biocompatibility and biodegradability [67].

## Stimuli-sensitive nanogels

### LHRH-targeted nanogels

Luteinizing hormone-releasing hormone (LHRH), also referred to as gonadotropin-releasing hormone (GnRH), is a decapeptide with the amino acid sequence pGlu-His-Trp-Ser-Tyr-Gly-Leu-Arg-Pro-Gly-NH₂ that plays a central role in regulating reproductive functions [68]. Beyond its physiological role in the pituitary gland, LHRH receptors (LHRH-R) are aberrantly expressed in a wide range of malignant tissues. Overexpression of the LHRH-R gene has been reported in breast, endometrial, ovarian, and prostate cancers, as well as in hormone-independent malignancies including glioblastoma, pancreatic cancer, lung carcinoma, and melanoma [69–70]. The high expression of LHRH-R in tumors has therefore attracted considerable interest for receptor-mediated targeted drug delivery. Previous studies have demonstrated that LHRH-R engagement may induce receptor-mediated internalization and cellular uptake, thereby enhancing the selective accumulation of therapeutic agents within tumor tissues [71]. Notably, several nanocarrier systems functionalized with LHRH ligands have been developed using conventional chemotherapeutic agents such as cisplatin. Although these systems do not constitute herbal nanogels, they provide important proof-of-concept evidence for the utility of LHRH-mediated targeting in cancer therapy [72]. The mechanistic insights gained from such studies are highly relevant to the rational design of herbal nanogel platforms, where LHRH ligands may similarly be employed to enhance the tumor-specific delivery of phytochemicals such as curcumin, resveratrol, berberine, and other bioactive compounds [73]. LHRH-targeted nanocarriers is intended to provide a translational framework for future receptor-targeted herbal nanogel systems rather than to present existing herbal Skin cancer therapy, LHRH-mediated targeting represents a promising strategy for enhancing the selective delivery of herbal nanogel formulations to tumor tissues. Recent research has increasingly focused on integrating LHRH-targeting ligands with nanometre-sized drug delivery systems (DDS), thereby improving tumor-specific accumulation and therapeutic efficacy [74]. Such targeted delivery approaches are particularly advantageous for phytochemical-loaded nanogels, as they enhance the bioavailability, stability, and cellular uptake of poorly soluble herbal bioactives while minimizing off-target toxicity [75]. Herbal nanogels incorporating bioactive phytoconstituents such as curcumin, quercetin, resveratrol, berberine, epigallocatechin gallate, and aloe-emodin have demonstrated considerable potential in skin cancer management due to their antioxidant, anti-inflammatory, anti-proliferative, and pro-apoptotic properties. Functionalization of these nanogels with LHRH analogues may further facilitate receptor-mediated uptake and targeted intracellular delivery to cancer cells overexpressing LHRH-R [76]. A modified PEG-derived polymethacrylic acid (PMAA) block copolymer, designated PEG-b-PMAA, was developed by Alam et al. using a polyethylene glycol linker compound [77]. Cisplatin-carrying copolymers were conjugated with [D-Lys6] GnRH. Nanogels suspended in PBS or plasma remained stable without aggregation for at least fifteen days while maintaining equilibrium [78]. The GnRH-R-expressing ovarian tumor carcinoma cell line A2780 exhibited 47% greater uptake of targeted nanogels after thirty minutes compared with non-targeted nanogels [79]. Furthermore, targeted nanogels generated higher intracellular concentrations of cisplatin and exhibited superior anticancer activity relative to non-targeted nanogels, whereas free cisplatin accumulated non-selectively [80]. Although the aforementioned system employs a synthetic chemotherapeutic agent, it provides valuable insights into receptor-mediated targeting strategies that can be translated to herbal nanogel formulations for skin cancer therapy. Similar targeting approaches may be employed to deliver phytochemicals selectively to melanoma and other cutaneous malignancies, thereby improving therapeutic efficacy while reducing systemic toxicity [81]. The GnRH-targeted nanogels produced tumor reduction of up to 75% in nude mice bearing human ovarian tumor xenografts, with sustained inhibition observed over twenty days [82]. In contrast, free cisplatin and non-targeted nanogels achieved a maximum tumor inhibition of approximately 50%. Notably, mice treated with targeted nanogels exhibited no significant changes in body weight [83]. The potential of ligand-mediated targeting strategies, such as LHRH functionalization, for the development of advanced herbal nanogel systems in skin cancer therapy. Future studies should focus on integrating receptor-targeted delivery with herbal bioactives to achieve enhanced therapeutic outcomes and improved translational applicability in melanoma and other skin cancers (Fig. 7) [84]. Receptor-targeted delivery systems offer exciting opportunities for enhancing the therapeutic efficacy of phytochemicals against skin cancer. Natural bioactive compounds such as curcumin, quercetin, resveratrol, berberine, epigallocatechin gallate, and genistein possess significant antiproliferative, antioxidant, anti-inflammatory, and pro-apoptotic activities; however, their clinical application is hindered by poor solubility and limited bioavailability [85]. Incorporation of these phytoconstituents into ligand-functionalized nanogels may facilitate selective accumulation within tumor tissues and enhance intracellular uptake. Particularly in melanoma, where LHRH receptors have been identified [86], LHRH-mediated targeting strategies may provide an effective approach for the localized delivery of herbal therapeutics. Functionalization of herbal nanogels with receptor-specific ligands can improve the retention and controlled release of phytochemicals at the tumor site while minimizing systemic toxicity. Such targeted systems combine the advantages of nanotechnology with the pharmacological potential of plant-derived compounds and may represent an emerging strategy for personalized skin cancer therapy [87]. Furthermore, the combination of receptor-mediated targeting with phytochemical-loaded nanogels has the potential to enhance therapeutic outcomes by increasing tumor specificity and reducing the adverse effects associated with conventional chemotherapeutic agents. Therefore, ligand-targeted herbal nanogels represent a promising next-generation platform for the treatment of melanoma and other skin malignancies, warranting further investigation for clinical translation [88].

**Table 3:** Stimuli-Responsive Herbal Nanogel Systems for Skin Cancer Therapy: Microenvironmental Relevance, Therapeutic Advantages, Translational Challenges, and Representative References

Responsive Nanogel Type	Stimulus/Trigger	Relevance to Skin Cancer Microenvironment	Therapeutic Advantages	Major Translational Challenges	Clinical Applicability in Skin Cancer	Ref
pH-Responsive Nanogels	Weakly acidic tumor pH (≈ 6.5–6.8)	Skin tumors exhibit mild extracellular acidity due to glycolytic metabolism and hypoxia	Site-specific drug release, reduced systemic toxicity, enhanced phytochemical retention	Limited pH gradient between tumor and healthy skin; inter-patient variability	Highly promising for melanoma and non-melanoma skin cancers	[89]
Enzyme-Responsive Nanogels	Matrix metalloproteinases (MMPs), hyaluronidase, lipases	Elevated enzyme activity in invasive skin tumors	Precise and controlled release within tumor tissues	Heterogeneous enzyme expression; off-target activation	Suitable for personalized therapy	[90]
Redox-Responsive Nanogels	Elevated glutathione (GSH) and ROS	Tumor cells exhibit abnormal redox homeostasis	Enhanced intracellular release and cytotoxicity	Variability in redox status; premature drug release	Effective for intracellular phytochemical delivery	[91]
Thermo-Responsive Nanogels	Local hyperthermia or temperature changes	External heating can be applied to skin lesions	Controlled release and synergistic photothermal effects	Risk of thermal damage; temperature control challenges	Applicable for localized skin tumors	[92]
Photo-Responsive Nanogels	UV, visible, or NIR light	Skin tumors are easily accessible to light irradiation	Non-invasive and spatiotemporal drug release	Limited penetration depth; phototoxicity	Particularly suitable for superficial melanoma	[93]
LHRH-Targeted Nanogels	LHRH receptor overexpression	LHRH-R is expressed in melanoma and other cancers	Enhanced receptor-mediated uptake and targeting	Receptor heterogeneity; ligand immunogenicity	Promising for precision oncology	[94]
Bacteria/Microbial Microenvironment-Responsive Nanogels	Bacterial enzymes, lipases, microbial metabolites	Tumor-associated microbiota may provide endogenous triggers	Selective release and prolonged retention	Safety concerns; variability in microbiota	Experimental stage with limited clinical translation	[95]
Magnetic-Responsive Nanogels	External magnetic field	Enables targeted localization to skin lesions	Non-invasive targeting and controlled release	Need for magnetic devices; nanoparticle toxicity	Feasible for localized treatment	[96]
Dual/Multi-Stimuli Responsive Nanogels	Combination of pH, enzyme, redox, or temperature	Mimics the complex skin tumor microenvironment	Superior targeting and therapeutic efficacy	Complex synthesis; scalability and regulatory issues	Represents the future of precision nanomedicine	[97]

GSH, glutathione; ROS, reactive oxygen species; MMPs, matrix metalloproteinases; LHRH-R, luteinizing hormone-releasing hormone receptor; NIR, near-infrared

### Nanogel sensitive to bacteria-accumulated tumor artificial environment

Although tumor-targeting bacteria have emerged as promising vectors for cancer-specific therapeutic delivery due to their inherent ability to selectively colonize tumor tissues and transport therapeutic cargos [98], their application in the context of herbal nanomedicine remains largely exploratory. In skin cancer therapy, particularly melanoma, the primary emphasis has increasingly shifted toward biocompatible nanogel platforms capable of delivering plant-derived bioactives with enhanced specificity and reduced systemic toxicity [99]. Bacteria-responsive nanogel systems reported to date have primarily been developed for the delivery of synthetic chemotherapeutic agents rather than herbal bioactives. Therefore, these systems are discussed in the present review as proof-of-concept examples of stimuli-responsive nanomedicine whose design principles may be adapted for future herbal nanogel formulations [100]. Their extensive gene-loading capability enables the simultaneous delivery of multiple therapeutic genes and the construction of specialized cellular communication pathways, thereby facilitating complex anticancer activities [101]. Therapeutic strategies are increasingly focused on the encapsulation of phytochemicals such as curcumin, quercetin, resveratrol, epigallocatechin gallate, and berberine, which possess intrinsic antioxidant, anti-inflammatory, and antiproliferative properties against skin cancer cells. Nanogel-mediated delivery of these herbal compounds enhances their stability, solubility, skin permeation, and sustained release, thereby improving therapeutic efficacy [102]. Despite their significant therapeutic potential, successful treatment of malignancies will likely require combinatorial approaches because of the molecular and histopathological heterogeneity of cancer. Consequently, engineered tumor-targeting microorganisms are anticipated to become valuable tools in cancer therapy, and their clinical applications are currently being explored [103]. Nevertheless, future translational research in skin cancer management is expected to increasingly integrate receptor-targeted and stimuli-responsive herbal nanogel systems, either alone or in combination with advanced biological approaches, to achieve enhanced therapeutic outcomes while minimizing adverse effects [104]. Herbal nanogel formulations, bacteria-assisted targeting strategies provide a novel approach for improving the site-specific delivery of phytochemicals used in skin cancer therapy. The selective accumulation of tumor-targeting bacteria within hypoxic and necrotic regions of tumors can be exploited to enhance the localized release of phytochemical-loaded nanogels [105]. Such systems may increase the concentration of herbal bioactive compounds at the tumor site while minimizing exposure to healthy tissues. Natural anticancer agents, including curcumin, quercetin, resveratrol, berberine, epigallocatechin gallate, and genistein, exhibit promising antiproliferative and pro-apoptotic activities against melanoma and non-melanoma skin cancers [106]. However, their poor solubility, instability, and low bioavailability limit their clinical application. Incorporation of these phytochemicals into bacteria-responsive nanogels offers a strategy to overcome these limitations and achieve controlled and targeted drug release [107]. Furthermore, bacterial enzymes and metabolites present within the tumor microenvironment can act as biological stimuli to trigger the degradation of polymeric matrices and promote the release of encapsulated herbal compounds. Such bacteria-responsive herbal nanogels can provide enhanced penetration into tumor tissues, prolonged retention, and improved therapeutic efficacy [108]. The integration of engineered bacteria with phytochemical-loaded nanogels represents an emerging multifunctional platform that combines biological targeting with nanotechnology-based drug delivery [109]. Although still in its early stages of development, this approach has the potential to improve the effectiveness of natural anticancer agents and may contribute to the development of safer and more personalized treatments for melanoma and other skin malignancies [110].

**Fig. 8:**
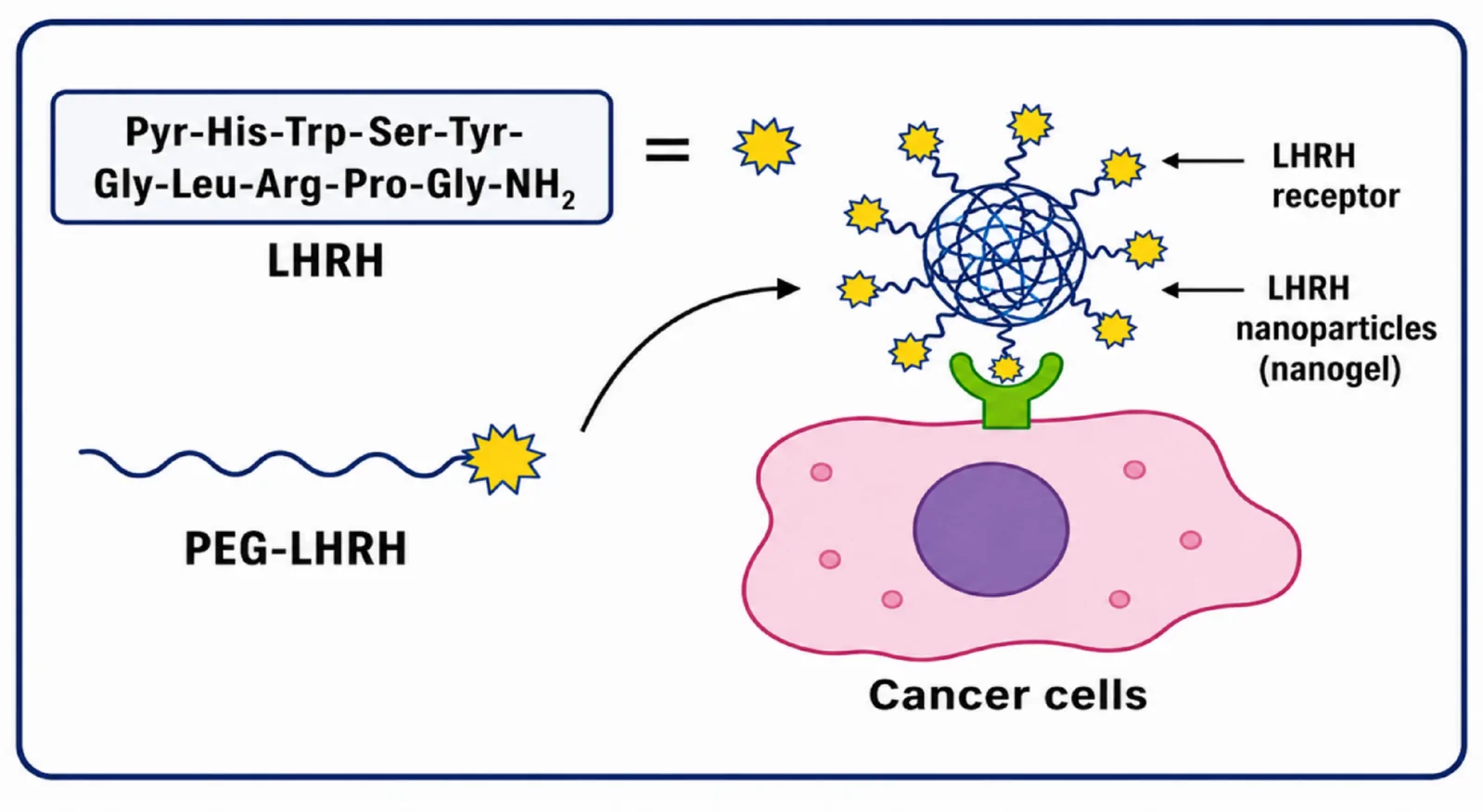
Schematic illustration of LHRH peptide-functionalized nanogels for targeted cancer therapy. The luteinizing hormone-releasing hormone (LHRH) peptide [Pyr-His-Trp-Ser-Tyr-Gly-Leu-Arg-Pro-Gly-NH₂] is conjugated to poly (ethylene glycol) (PEG) to form PEG-LHRH, which is subsequently coupled to nanogel nanoparticles [111]. The resulting LHRH-decorated nanogels selectively recognize and bind to overexpressed LHRH receptors on cancer cells, facilitating receptor-mediated cellular uptake and enhancing targeted drug delivery

**Fig. 9:**
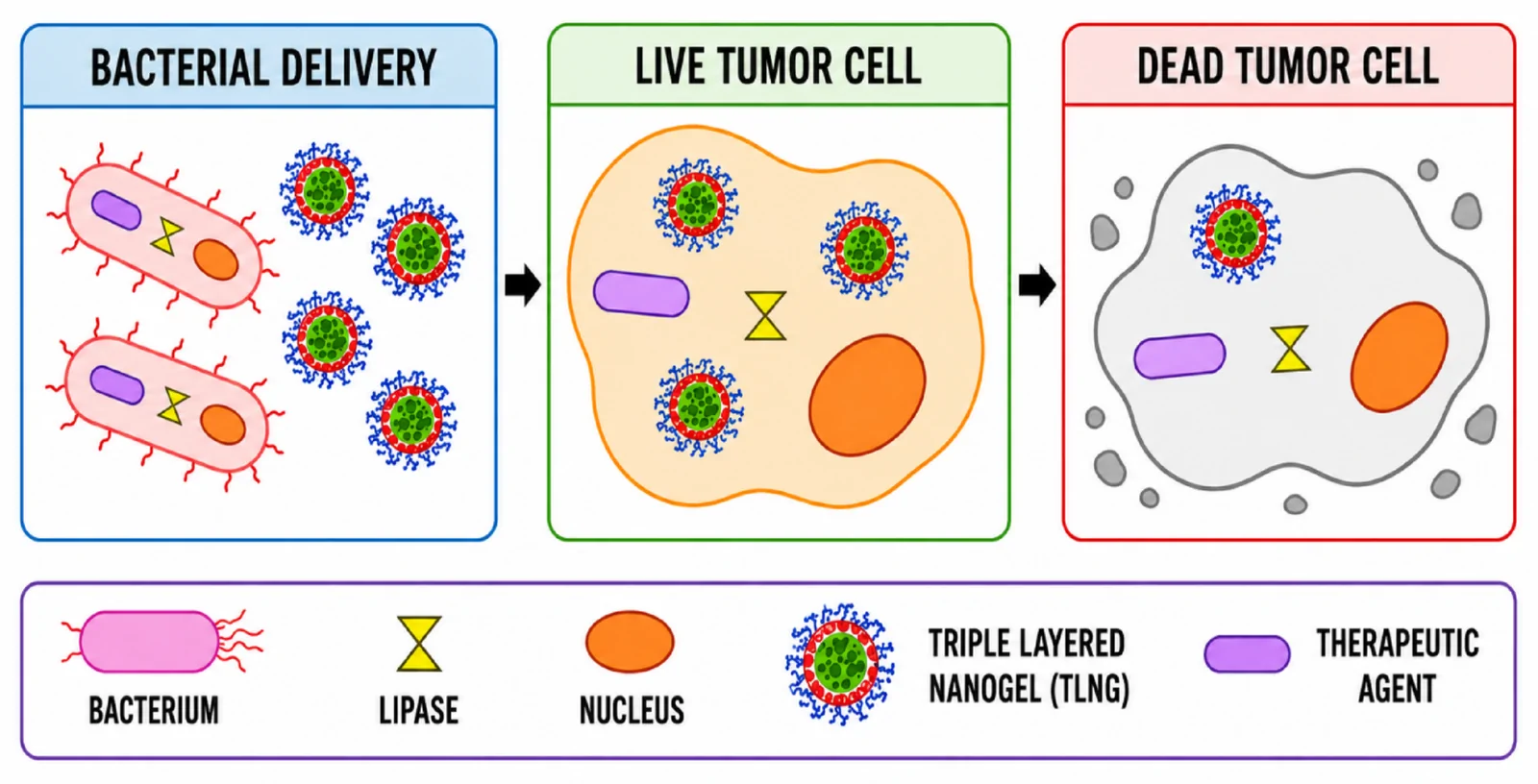
Schematic illustration of a nanogel responsive to a bacteria-laden tumor artificial microenvironment. After bacterial targeting to the tumor site, the lipase-rich environment activates and degrades the triple-layered nanogel (TLNG) and allows sustained release of the therapeutic agent inside the living tumor cells, leading to tumor cell death

Figure 8: Illustrative representation of LHRH peptide-decorated nanogel for cancer cell targeting. The LHRH peptide (Pyr-His-Trp-Ser-Tyr-Gly-Leu-Arg-Pro-Gly-NH₂) is attached to poly (ethylene glycol) (PEG) to create PEG-LHRH, which is then coupled to nanogel nanoparticles. These LHRH-functionalized nanogels target cancer cells via LHRH receptors, which are often overexpressed on cancer cells, allowing for targeted cellular uptake [112]. Li et al. [78]. developed an innovative strategy for selective anticancer drug delivery based on bacteria-sensitive triple-layered nanogels (TLNs). In this approach, bacteria-responsive triple-layered nanogels served as carriers for targeted drug delivery within bacteria-accumulated tumor tissues [113]. Conventional chemotherapeutic agents rather than plant-derived compounds; thus, it does not represent a true herbal nanogel system. Nevertheless, the bacteria-responsive mechanism demonstrated by this platform provides valuable insights for the future development of phytochemical-loaded nanogels in skin cancer therapy [114]. Although this platform was originally designed for the delivery of synthetic chemotherapeutics, its underlying principles may be adapted for the targeted delivery of herbal bioactives in skin cancer therapy. Phytochemicals such as curcumin, resveratrol, quercetin, berberine, and epigallocatechin gallate have demonstrated significant anticancer potential against melanoma through modulation of oxidative stress, inflammation, apoptosis, and cell proliferation pathways [115]. *Staphylococcus aureus* NCTC8325 SBY1 was employed as an experimental model to establish a bacteria-enriched artificial tumor microenvironment for evaluating the responsiveness of doxorubicin-loaded triple-layered nanogels (TLND) [116]. However, the deliberate systemic administration of live bacteria poses substantial safety concerns, including infection risk, immune activation, and potential sepsis, which may restrict its clinical translation for skin cancer applications [117]. Consequently, current research is increasingly focused on safer alternatives, such as bacterial enzymes, bacterial membrane vesicles, or tumor-associated microbial signatures, to trigger localized drug release within the skin tumor microenvironment without introducing viable microorganisms. As a result, microorganisms surrounding the tumor preferentially degraded TLND, leading to selective release of the chemotherapeutic agent and targeted destruction of cancer cells [118]. Such bacteria-responsive systems may offer enhanced therapeutic specificity in skin cancer when activated by endogenous microbial cues or non-viable bacterial components. The combination of bacteria-responsive TLND and SBY1 enhanced both the specificity and therapeutic efficacy of the chemotherapeutic treatment [119]. Bacteria-responsive nanogels may similarly be engineered to encapsulate plant-derived compounds, enabling controlled and stimuli-responsive release specifically within the tumor microenvironment while minimizing off-target toxicity [120]. Although doxorubicin-based formulations fall outside the scope of herbal nanomedicine, the underlying stimuli-responsive release strategy may be translated to herbal therapeutics. Specifically, phytochemicals such as curcumin, resveratrol, quercetin, and berberine could potentially be incorporated into similar bacteria-responsive nanogel matrices to achieve tumor-specific delivery and controlled release [121]. Furthermore, this strategy has the potential to be extended to various synthetic tumor microenvironments and environmentally responsive nanomaterials. Following intravenous administration, attenuated SBY1 bacteria preferentially accumulated in tumor tissues and generated a unique bacteria-enriched microenvironment [122]. These bacteria were simultaneously eliminated from normal tissues within a short period. Consequently, the bacteria-colonized tumor microenvironment selectively triggered the release of doxorubicin from TLND, resulting in efficient tumor cell destruction [123]. Such tumor-responsive systems highlight the broader potential of integrating environmental stimuli with herbal nanogel formulations for melanoma therapy. By improving the stability, bioavailability, and targeted delivery of phytochemicals, stimuli-responsive herbal nanogels may represent a safer and more effective alternative to conventional chemotherapy in skin cancer management [124] (Fig. 9). Bacteria-responsive systems represent an emerging approach for the site-specific delivery of phytochemicals against skin cancer. The enzyme-rich and bacteria-associated tumor microenvironment can serve as an internal trigger for the controlled degradation of polymeric nanogel matrices and subsequent release of encapsulated herbal constituents [124]. Such systems offer the possibility of improving the therapeutic index of natural anticancer agents while reducing systemic toxicity. Several phytochemicals, including curcumin, quercetin, resveratrol, berberine, epigallocatechin gallate, and genistein, have demonstrated significant anticancer activities through modulation of oxidative stress, apoptosis, angiogenesis, and inflammatory signaling pathways [125]. However, their poor bioavailability and rapid degradation limit their clinical effectiveness. Encapsulation within bacteria-responsive nanogels provides a promising strategy to enhance their stability and ensure localized drug release within tumor tissues. In melanoma and non-melanoma skin cancers, bacteria-responsive herbal nanogels may facilitate prolonged retention and selective accumulation of phytochemicals at the tumor site. Bacterial enzymes and metabolites present within the tumor microenvironment can act as biological stimuli that trigger the release of encapsulated phytoconstituents, thereby increasing therapeutic efficacy and minimizing off-target effects 126]. Moreover, the integration of bacterial targeting with herbal nanogel technology combines the advantages of biological specificity and natural-product-based therapy. Such multifunctional systems have the potential to overcome the limitations associated with conventional chemotherapy and may provide safer and more effective treatment options for skin cancer [127]. Although still in the early stages of development, bacteria-responsive herbal nanogels represent a promising area of research in precision nanomedicine and warrant further investigation for clinical translation [128]. Bacteria-responsive nanogels should be regarded as an emerging translational strategy rather than an established category of herbal nanogels. Their inclusion aims to provide mechanistic insights and future directions for integrating biological targeting approaches with phytochemical-based nanomedicine in skin cancer therapy [129].

### Acid-activating prodrug nanogels

Filho et al. [84]. reported the rapid fabrication of endosomal pH-responsive doxorubicin (DOX)-based prodrug nanogels. These nanogels were synthesized using poly (ethylene glycol) and poly (2-hydroxyethyl methacrylate-co-glycine methacrylamide-DOX) [85]. Although these formulations were designed for the delivery of synthetic chemotherapeutic agents, they provide an important framework for the development of herbal nanogel systems aimed at improving the therapeutic efficacy of phytoconstituents against skin cancer [86]. Herbal compounds such as curcumin, resveratrol, quercetin, berberine, and epigallocatechin gallate possess significant anticancer properties but often suffer from poor aqueous solubility, limited bioavailability, and rapid degradation, which can be effectively addressed through nanogel encapsulation [87]. A distinctive feature of these prodrug nanogels is their ability to combine the advantages of nanogels with those of macromolecular therapeutics. These formulations exhibit excellent water dispersibility, minimal drug leakage under physiological conditions, and rapid release of encapsulated drugs under mildly acidic conditions or within intracellular compartments [130]. Such pH-responsive characteristics are particularly advantageous for herbal nanogels, as the acidic tumor microenvironment of melanoma and other skin cancers can trigger site-specific release of phytochemicals, thereby maximize therapeutic efficacy while reduce systemic toxicity [131]. In addition, their nanoscale dimensions and stealth characteristics contribute to prolonged blood circulation and enhanced tumor accumulation. Although previous studies have demonstrated potent cytotoxic activity of DOX-loaded nanogels in non-skin cancer cell lines such as MCF-7 and RAW 264.7 cells [132], these findings underscore the broader therapeutic potential of nanogel-based drug delivery systems. In the context of skin cancer, similar nanogel platforms may facilitate enhanced drug accumulation and therapeutic efficacy within the skin tumor microenvironment. Compared with their corresponding macromolecular drug conjugates, the nanogels exhibited nearly ten-fold lower IC50 values. Similarly, herbal nanogels encapsulating natural anticancer agents have demonstrated enhanced cellular uptake, sustained release, and improved cytotoxic effects against skin cancer cells in preclinical investigations, highlighting their potential as safer alternatives to conventional chemotherapy [133]. Furthermore, PEG-b-P(HEMA-co-GMA-hydrazide) [88] and its polymeric carrier were found to be non-toxic at concentrations up to 1.32 mg/mL. Therefore, these DOX-containing prodrug nanogels have shown significant potential for tumor-targeted drug delivery applications [134]. The design principles of these stimuli-responsive nanogels may be translated to plant-based formulations, thereby facilitating the development of biocompatible and targeted herbal nanogels for melanoma therapy and other skin malignancies [135] (Fig. 9). The acidic tumor microenvironment associated with melanoma and non-melanoma skin cancers provides an attractive opportunity for the development of pH-responsive herbal nanogels. Such systems are capable of exploiting endosomal and lysosomal acidity to trigger the selective release of encapsulated phytochemicals directly within cancer cells, thereby enhancing therapeutic efficacy while minimizing systemic toxicity [136]. Natural compounds such as curcumin, quercetin, resveratrol, berberine, epigallocatechin gallate, genistein, and aloe vera-derived constituents possess remarkable antioxidant, anti-inflammatory, antiproliferative, and pro-apoptotic properties. However, their clinical application is limited by poor aqueous solubility, rapid metabolism, and low bioavailability. Encapsulation of these phytochemicals within pH-sensitive nanogel systems offers a promising strategy to overcome these limitations and improve their therapeutic performance. Biocompatible polymers including PEG, PLGA, chitosan, hyaluronic acid, and dextran have been extensively investigated for the fabrication of pH-responsive herbal nanogels [137]. These systems provide enhanced stability, sustained drug release, improved skin penetration, and prolonged retention of phytochemicals at tumor sites [137]. Furthermore, controlled release under acidic conditions enables selective accumulation of herbal bioactive compounds within malignant tissues while reducing damage to normal cells. Consequently, pH-responsive herbal nanogels represent an emerging platform for targeted skin cancer therapy. The combination of stimulus-responsive nanotechnology with naturally derived anticancer agents has the potential to provide safer and more effective alternatives to conventional chemotherapy and may facilitate the clinical translation of phytochemical-based treatments for melanoma and other skin malignancies [138].

**Fig. 10:**
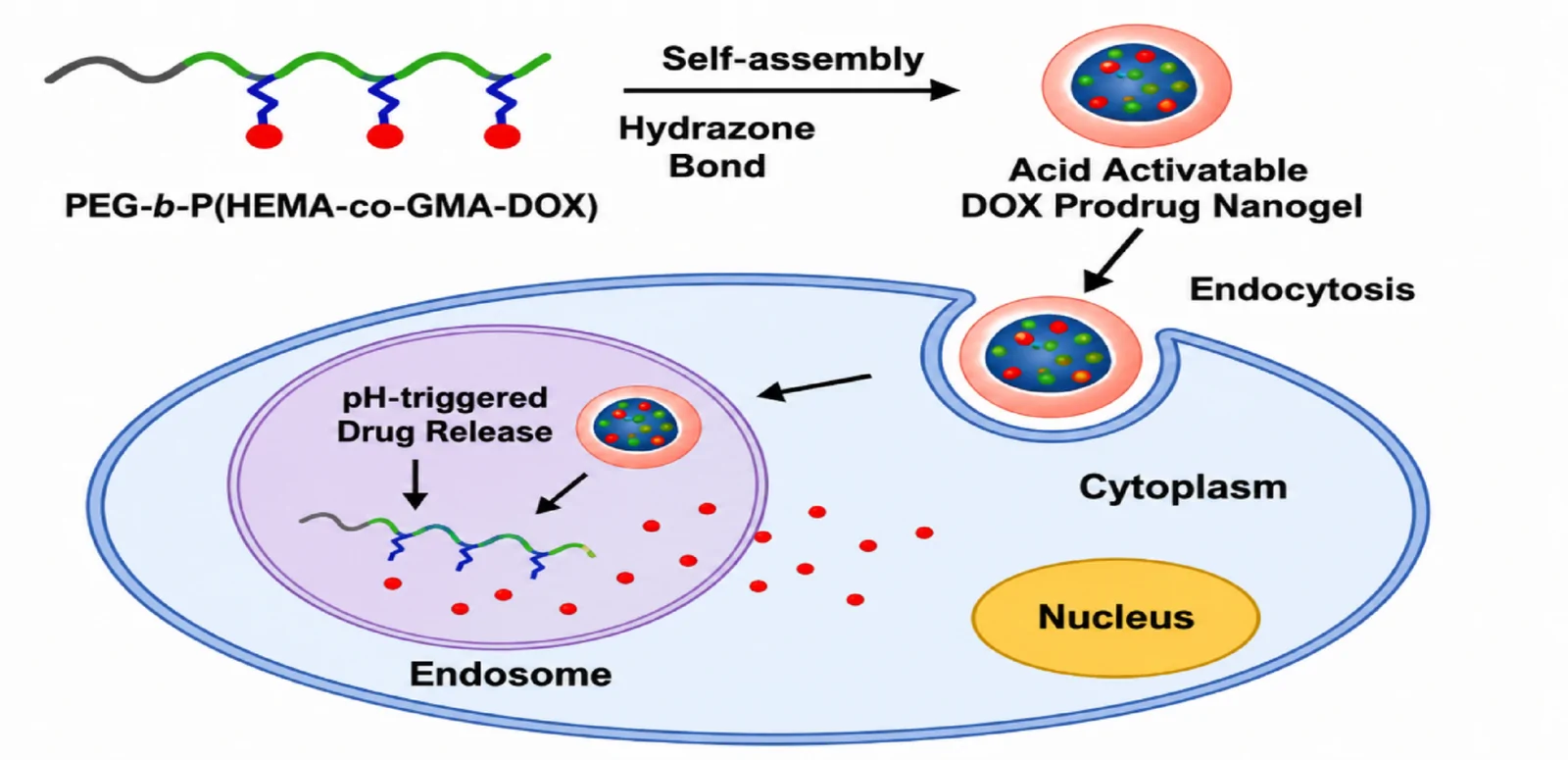
Schematic representation of acid-responsive prodrug nanogels comprising PEG-b-P(HEMA-co-GMA-DOX) with hydrazone linkages. The amphiphilic polymer spontaneously self-assembles into nanogels loaded with doxorubicin (DOX). Cellular uptake of nanogels occurs via endocytosis, and they are subsequently located in endosomes. The acidic environment of the endosomes leads to the cleavage of the hydrazone bond and pH-responsive drug delivery. The DOX molecules are then released into the cytoplasm and eventually into the nucleus for targeted cancer treatment

### Adamantane-doxorubicin prodrug in pH-responsive polysaccharide capsules

A pH-modulated drug delivery strategy was achieved through the development of specialized supramolecular microcapsules (SMCs) possessing layered structures loaded with therapeutic agents on their outer surfaces [139]. These SMCs were fabricated using a layer-by-layer approach based on the self-assembly of dextran-derived polyaldehyde-graft-adamantane (PAD-g-AD) and carboxymethyl dextran-graft-cyclodextrin (CMD-g-CD) on calcium carbonate particles through host-guest interactions [140]. Although these systems were primarily designed for the delivery of synthetic drugs, their structural design provides valuable insights for the development of herbal nanogel formulations intended for skin cancer therapy. Natural phytoconstituents such as curcumin, quercetin, resveratrol, berberine, and epigallocatechin gallate exhibit potent anticancer activities; however, their clinical application is often hindered by poor solubility, instability, and limited bioavailability, which can be effectively overcome using stimuli-responsive nanocarriers [141]. Simultaneously, adamantane-modified doxorubicin (AD-Dox) was immobilized on the microcapsule walls through the same host-guest interaction mechanism. Drug release studies conducted under various pH conditions demonstrated that mildly acidic environments promoted cleavage of pH-sensitive hydrazone bonds connecting the adamantane moieties to PAD or Dox, resulting in SMC disintegration and drug release [142]. This pH-responsive behaviour is particularly relevant for melanoma and other skin cancers, as the acidic tumor microenvironment can trigger selective release of encapsulated herbal bioactives, thereby enhancing therapeutic efficacy while minimizing systemic adverse effects [92]. This pH-controlled release behaviour enhances intracellular drug uptake and improves therapeutic efficacy against cancer cells [93]. Accordingly, a novel class of supramolecular microcapsules was developed for pH-responsive drug delivery applications [94]. Analogously, herbal nanogels engineered with pH-sensitive linkages can achieve sustained and site-specific delivery of phytochemicals, improving their cellular uptake and antitumor activity against skin cancer cells [143]. Drug release studies revealed that these SMCs readily degraded under weakly acidic conditions owing to the cleavage of hydrazone bonds, thereby facilitating rapid release of the encapsulated drug [144]. In vitro studies further demonstrated that drug-loaded SMCs exhibited lower cytotoxicity under physiological conditions and enhanced cytotoxic activity under acidic conditions [145]. Similarly, pH-responsive herbal nanogels have demonstrated the capacity to improve the stability, skin permeation, and controlled release of natural anticancer agents, thereby maximizing their therapeutic benefits while reducing toxicity to healthy tissues [146]. Their tumor-triggered release characteristics suggest significant potential for cancer treatment [147]. Overall, the principles underlying these supramolecular systems can be translated to the design of advanced herbal nanogel formulations for melanoma and other skin cancers, thereby supporting the development of safer, biocompatible, and targeted nanomedicine platforms [148]. Supramolecular pH-responsive systems provide an attractive platform for the controlled delivery of phytochemicals against melanoma and non-melanoma skin cancers. The acidic tumor microenvironment can act as an endogenous trigger for the degradation of supramolecular nanogel matrices, enabling selective release of plant-derived bioactive compounds at the tumor site [148]. Natural compounds such as curcumin, quercetin, resveratrol, berberine, epigallocatechin gallate, genistein, and aloe vera constituents possess considerable anticancer activities; however, their therapeutic application is limited by poor solubility, instability, and low bioavailability [149]. Encapsulation within supramolecular nanogel systems can improve their aqueous dispersibility, protect them from premature degradation, and facilitate sustained and targeted release [150]. Dextran and cyclodextrin-based supramolecular assemblies are particularly suitable for the incorporation of hydrophobic phytochemicals because of their excellent biocompatibility and host-guest interaction capabilities. Such systems can enhance cellular uptake and increase the accumulation of herbal bioactive compounds within tumor tissues, thereby improving therapeutic outcomes while reducing systemic toxicity [151]. Moreover, the pH-responsive behavior of supramolecular herbal nanogels enables selective release of phytoconstituents within acidic intracellular compartments such as endosomes and lysosomes [152]. This feature promotes apoptosis and inhibits tumor progression, making these systems promising candidates for targeted skin cancer therapy. Therefore, supramolecular pH-responsive herbal nanogels represent an emerging strategy that combines the advantages of nanotechnology and natural-product-based therapeutics for the management of skin cancers [153].

### Multifunctional chitin nanogels

**Fig. 11:**
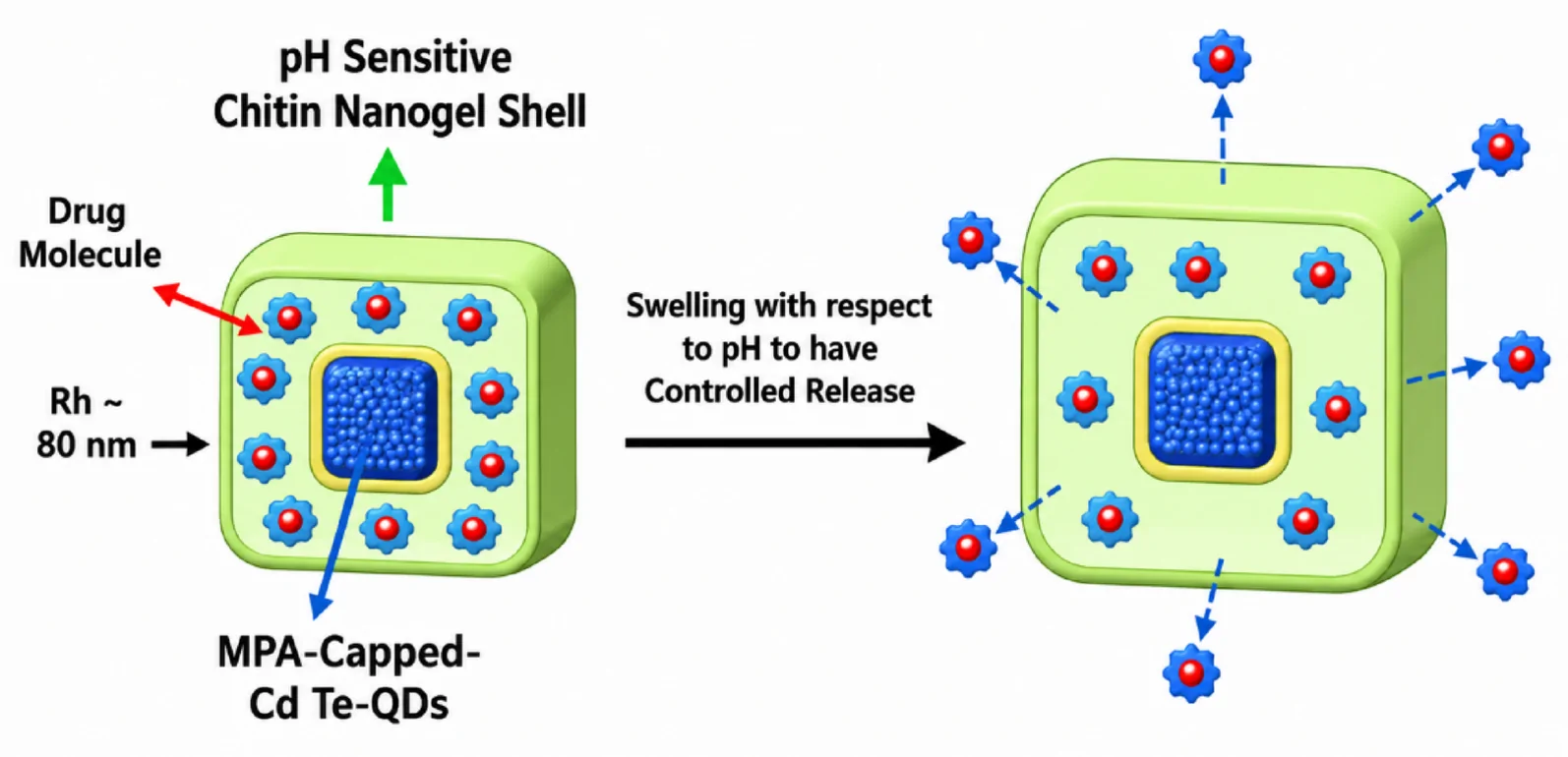
Diagram A schematic depicting the structure and function of multifunctional bovine serum albumin (BSA)-loaded CdTe quantum dots (QDs)-chitin nanogels (BSA-QD-CNGs). The design consists of a pH-responsive chitin nanogel shell that incorporates MPA-capped CdTe quantum dots and bovine serum albumin (BSA), with an average diameter of ~ 80 nm. The nanogel swells in response to changes in pH, facilitating sustained release of the drug molecules. pH-sensitive characteristic underscores the versatility of BSA-QD-CNGs for a wide range of biomedical applications such as targeted drug delivery, bioimaging, and controlled release of therapeutic drugs

Chitin Nano biomaterials have recently emerged as promising candidates in biotechnology owing to their potential applications in drug delivery and tissue engineering [96]. Advances in nanotechnology have enabled the integration of multiple functionalities into a single nanoparticle system [98]. Skin cancer therapy, naturally derived polymers such as chitin and chitosan have attracted considerable interest as nanogel matrices for the delivery of herbal bioactives due to their excellent biocompatibility, biodegradability, and mucoadhesive properties [98]. Quantum dots (QDs), owing to their unique optical properties, including broad excitation spectra, narrow emission profiles, high quantum yields, and excellent photostability, have been proposed as imaging and labelling tools for biomedical applications [99]. Although QD-based systems provide valuable imaging capabilities, their role in herbal nanomedicine is primarily supportive, enabling real-time tracking and monitoring of therapeutic responses rather than serving as the principal therapeutic component [99]. Surface conjugation of biopolymers with QDs facilitates the integration of imaging and therapeutic functions. Nanogels possess several desirable characteristics, including adjustable particle size, high surface area suitable for multivalent bioconjugation, high drug-loading capacity, controlled release behavior, and responsiveness to environmental stimuli, making them ideal candidates for drug delivery systems [100]. These properties are particularly advantageous for encapsulating phytochemicals such as curcumin, quercetin, resveratrol, berberine, and epigallocatechin gallate, which exhibit potent anticancer activities against melanoma but are limited by poor aqueous solubility and bioavailability [100]. Chitin and other naturally occurring polymers exhibit excellent biocompatibility and prolonged functional stability, rendering them highly suitable for biomedical applications. Their ability to interact with various ligands contributes to their widespread utilization. Chitin, composed of β-(1→4)-linked N-acetylglucosamine units, serves as the primary precursor for chitosan [101]. Chitin-based nanogels can therefore serve as efficient carriers for herbal compounds, facilitating sustained release, enhanced skin permeation, and targeted delivery to skin tumor tissues [101]. Brash et al. reported the synthesis of a novel generation of chitin nanogels (CNGs) with particle sizes below 100 nm using bovine serum albumin (BSA) as a model protein and demonstrated their multifunctionalization potential [102]. In their study, CNGs were conjugated with CdTe quantum dots (3.2–3.8 nm) through in situ immobilization within the chitin network, resulting in nanogels exhibiting the combined characteristics of both components [102] (Fig. 10). The theranostics potential of QD-conjugated nanogels, future research may replace protein cargos with plant-derived anticancer agents to develop multifunctional herbal nanogel systems for melanoma diagnosis and therapy [102]. Chitin is distinguished from other polysaccharides because of its abundance and its ability to interact with polyelectrolytes, making it valuable for numerous biological and technological applications [103]. The presence of multiple hydroxyl and acetamide groups enables further cross-linking with Cd2 + ions, thereby facilitating the incorporation of CdTe QDs within the gel matrix [104]. Moreover, these functional groups provide versatile sites for the encapsulation and controlled release of phytochemicals, thereby broadening the applicability of chitin-based nanogels in herbal skin cancer therapy [104]. The resulting multifunctionalized chitin-based nanogels have demonstrated considerable potential for simultaneous diagnosis, therapy, and monitoring of pharmacological responses in skin cancer management [105]. These nanogels are capable of integrating pH sensing, cellular imaging, and environmentally triggered drug delivery, thereby enabling real-time monitoring and targeted therapeutic intervention within the skin tumor microenvironment. Multifunctional chitin hybrid nanogels can be fabricated in aqueous media by incorporating MPA-capped CdTe QDs into the chitin matrix and subsequently loading them with BSA to form BSA-QD-CNG systems [106]. Such hybrid nanogels exhibit excellent stability, reversible pH-responsive behavior, controlled protein release, and high cytocompatibility, making them promising candidates for theranostics applications in skin cancers, including melanoma and non-melanoma skin malignancies [107]. Herbal nanomedicine, these multifunctional features can be exploited to develop theranostics herbal nanogels capable of simultaneous delivery of phytoconstituents and monitoring of therapeutic responses in melanoma and other skin cancers [107]. Furthermore, QD-conjugated nanogels are capable of penetrating cells within acidic pathological environments (pH 5–7.4), allowing pH sensing and controlled protein release while facilitating therapeutic monitoring [108]. Such stimuli-responsive systems may provide advanced platforms for the targeted delivery of herbal agents while enabling real-time assessment of treatment efficacy, thereby supporting translational applications in skin cancer nanomedicine [108]. Herbal nanogel formulations, chitin and its derivative chitosan represent highly attractive natural polymeric carriers because of their biodegradability, biocompatibility, mucoadhesive properties, and ability to enhance skin penetration. These characteristics make them particularly suitable for the encapsulation and localized delivery of plant-derived bioactive compounds for the treatment of melanoma and non-melanoma skin cancers [154]. Several phytochemicals, including curcumin, quercetin, resveratrol, berberine, epigallocatechin gallate, genistein, and aloe vera constituents, exhibit potent antioxidant, anti-inflammatory, antiproliferative, and pro-apoptotic activities. However, their clinical utility is limited by poor aqueous solubility, instability, and low bioavailability [155]. Chitin- and chitosan-based nanogels can overcome these limitations by providing enhanced stability, sustained drug release, and improved penetration through the skin barrier. Moreover, the multifunctional nature of chitin hybrid nanogels enables the simultaneous integration of imaging and therapy (theranostics) [156]. Quantum dot-conjugated chitin nanogels loaded with herbal phytoconstituents may provide real-time monitoring of drug distribution and treatment response while ensuring site-specific delivery of natural anticancer agents. Such systems offer the possibility of combining diagnosis and therapy within a single platform, thereby improving therapeutic precision and reducing systemic toxicity [157]. The pH-responsive behavior of chitin-based nanogels is particularly advantageous in skin cancer treatment, as the acidic tumor microenvironment can trigger selective release of encapsulated phytochemicals [158]. Consequently, multifunctional chitin-based herbal nanogels represent a promising theragnostic platform that combines natural polymeric materials, phytomedicines, and imaging technologies for the development of advanced and personalized skin cancer therapies [159]. Figure 11: Schematic representation of multifunctional bovine serum albumin (BSA)-loaded CdTe quantum dot (QD)-chitin nanogels (BSA-QD-CNGs). The nanoplatform comprises a pH-responsive chitin nanogel matrix encapsulating mercaptopropionic acid (MPA)-capped CdTe quantum dots and bovine serum albumin (BSA), with an average particle diameter of approximately 80 nm. The chitin-based nanogel undergoes reversible swelling in response to variations in pH, enabling sustained and controlled release of therapeutic cargo. In addition to facilitating drug delivery, the incorporated CdTe quantum dots impart fluorescence properties for bioimaging applications [160]. Owing to their pH-sensitive behavior and multifunctional design, BSA-QD-CNGs represent a versatile nanocarrier system with significant potential for targeted drug delivery, controlled therapeutic release, and biomedical imaging.

### Triple-layered polymeric nanogel with lipase sensitivity

**Fig. 12:**
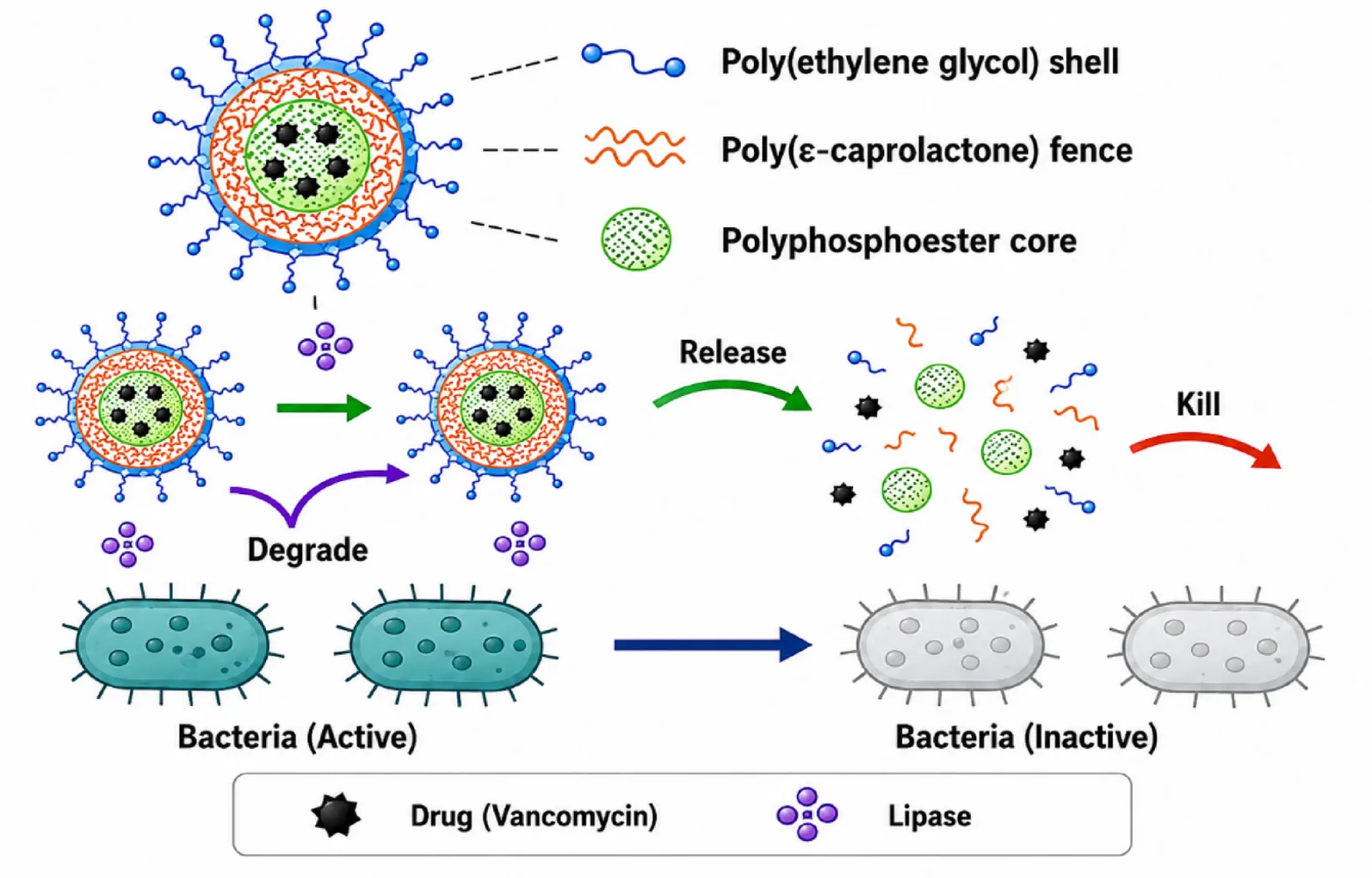
On-demand drug delivery using bacterial lipase for the treatment of bacterial infections with a polymeric triple-layered nanogel (TLN). The nanogel comprises a cross-linked polyphosphoester core, a lipase-degradable poly(ε-caprolactone) (PCL) intermediate layer, and an exterior layer of poly (ethylene glycol) (PEG). The PCL interlayer is degraded by bacterial lipase, which results in on-demand release of the encapsulated drug. The drug targets active bacteria, leading to their destruction and showcasing a responsive approach to combating bacterial infections

One of the major challenges in nanomedicine is the site-specific delivery of therapeutic agents to diseased tissues [160]. Nanoparticles are capable of adapting to the microenvironment of the target site while encapsulating therapeutic compounds, thereby minimizing premature drug leakage and ensuring controlled drug release [161]. A variety of internal and external stimuli, including pH, redox potential, temperature, enzyme activity, competitive chemical interactions, photoirradiation, and magnetic fields, have been exploited to activate responsive drug delivery systems [162]. Nanoparticles can be fabricated from numerous materials, including magnetic nanoparticles, multilayered cross-linked micelles, and mesoporous silica particles [163]. Most of these systems are responsive to the extracellular and intracellular tumor microenvironment, allowing them to function as passive or active targeting systems for cancer therapy [164]. Although many stimuli-responsive nanocarriers have been developed for the delivery of conventional chemotherapeutic agents, their design principles are increasingly being explored for the encapsulation and targeted delivery of plant-derived bioactives [165]. Accordingly, the following discussion on lipase-sensitive nanogels is included to provide mechanistic insights relevant to the future development of herbal nanogel systems for skin cancer therapy. Considerable efforts have also been directed toward the development of advanced antimicrobial delivery systems [165]. Liposomes, polymeric nanoparticles, nanosuspensions, and conjugates have demonstrated the potential to improve pharmacokinetics and therapeutic efficacy [166]. In 2012, Kaur et al. developed a novel bacteria-responsive delivery system based on a triple-layer nanogel (TLN) containing a bacterial lipase-sensitive poly(ε-caprolactone) (PCL) interlayer [118]. Lipase-sensitive TLN platform was originally designed for the delivery of synthetic therapeutic agents rather than herbal compounds and therefore does not represent a true herbal nanogel system. Nevertheless, its enzyme-responsive architecture provides a valuable proof-of-concept for engineering phytochemical-loaded nanogels with site-specific and stimuli-responsive drug release capabilities. In this system, the PCL intermediate layer is positioned between a poly (ethylene glycol) shell and a cross-linked polyphosphoester core (Fig. 12). The compact PCL barrier prevents premature drug leakage and protects the encapsulated therapeutic agents before reaching the site of infection [167], thereby minimizing adverse effects associated with uncontrolled drug release [167]. Bacterial lipases, which are abundantly secreted by numerous pathogenic microorganisms, subsequently hydrolyse the PCL barrier and trigger drug release [168]. Because lipase production has been implicated as a virulence factor in several bacterial species, it has been investigated as a biological trigger for controlled drug delivery [169]. Accordingly, degradation of the hydrophobic PCL layer facilitates on-demand release of therapeutic agents and suppresses microbial proliferation. Herbal nanomedicine, similar lipase-responsive nanogel systems could potentially be adapted to encapsulate phytochemicals such as curcumin, resveratrol, quercetin, berberine, and epigallocatechin gallate, thereby improving their stability, bioavailability, and tumor-specific delivery in skin cancer therapy [170]. A polymeric triple-layer nanogel system with spontaneous release characteristics was fabricated using a simple “arm-first synthesis” strategy [171]. The incorporation of a lipase-sensitive PCL interlayer enabled enzyme-triggered release of encapsulated drugs [172]. In the absence of lipase-producing bacteria, negligible drug leakage was observed, whereas rapid drug release occurred when lipase or lipase-secreting microorganisms were present. These findings demonstrated that nanogel-mediated delivery effectively inhibited extracellular bacterial growth [173]. Stimuli-responsive systems provide an attractive platform for the targeted delivery of phytochemicals in melanoma and non-melanoma skin cancers. The tumor microenvironment exhibits unique biochemical characteristics, including acidic pH, elevated enzyme concentrations, oxidative stress, and altered redox states, which can serve as endogenous triggers for the selective release of encapsulated herbal compounds [174. Natural anticancer agents such as curcumin, quercetin, resveratrol, berberine, epigallocatechin gallate, genistein, and aloe vera-derived constituents possess remarkable antiproliferative, antioxidant, anti-inflammatory, and pro-apoptotic activities [175]. However, their poor aqueous solubility and low bioavailability limit their clinical application. Encapsulation within stimuli-responsive nanogels can overcome these limitations by enhancing stability, protecting phytochemicals from degradation, and enabling controlled drug release at the tumor site. Enzyme-responsive herbal nanogels represent a particularly promising strategy because enzymes overexpressed within the tumor microenvironment can selectively degrade polymeric matrices and trigger localized release of phytoconstituents [176]. Similarly, pH-responsive and redox-responsive herbal nanogels exploit tumor-associated biochemical changes to achieve site-specific delivery while minimizing systemic toxicity. Natural polymers such as chitosan, dextran, hyaluronic acid, gelatin, and alginate have been extensively explored for the fabrication of multifunctional herbal nanogels owing to their excellent biocompatibility and biodegradability [177]. These systems facilitate enhanced skin penetration, prolonged retention, and sustained release of phytochemicals, thereby improving therapeutic efficacy against skin cancers. Consequently, stimuli-responsive herbal nanogels represent an emerging class of intelligent drug delivery systems that combine the advantages of nanotechnology and phytotherapy. Such multifunctional formulations hold considerable promise for improving the safety, specificity, and therapeutic outcomes of skin cancer treatment and may contribute significantly to the future development of personalized nanomedicine [178].

### PLGA nanogel for drug delivery

**Fig. 13:**
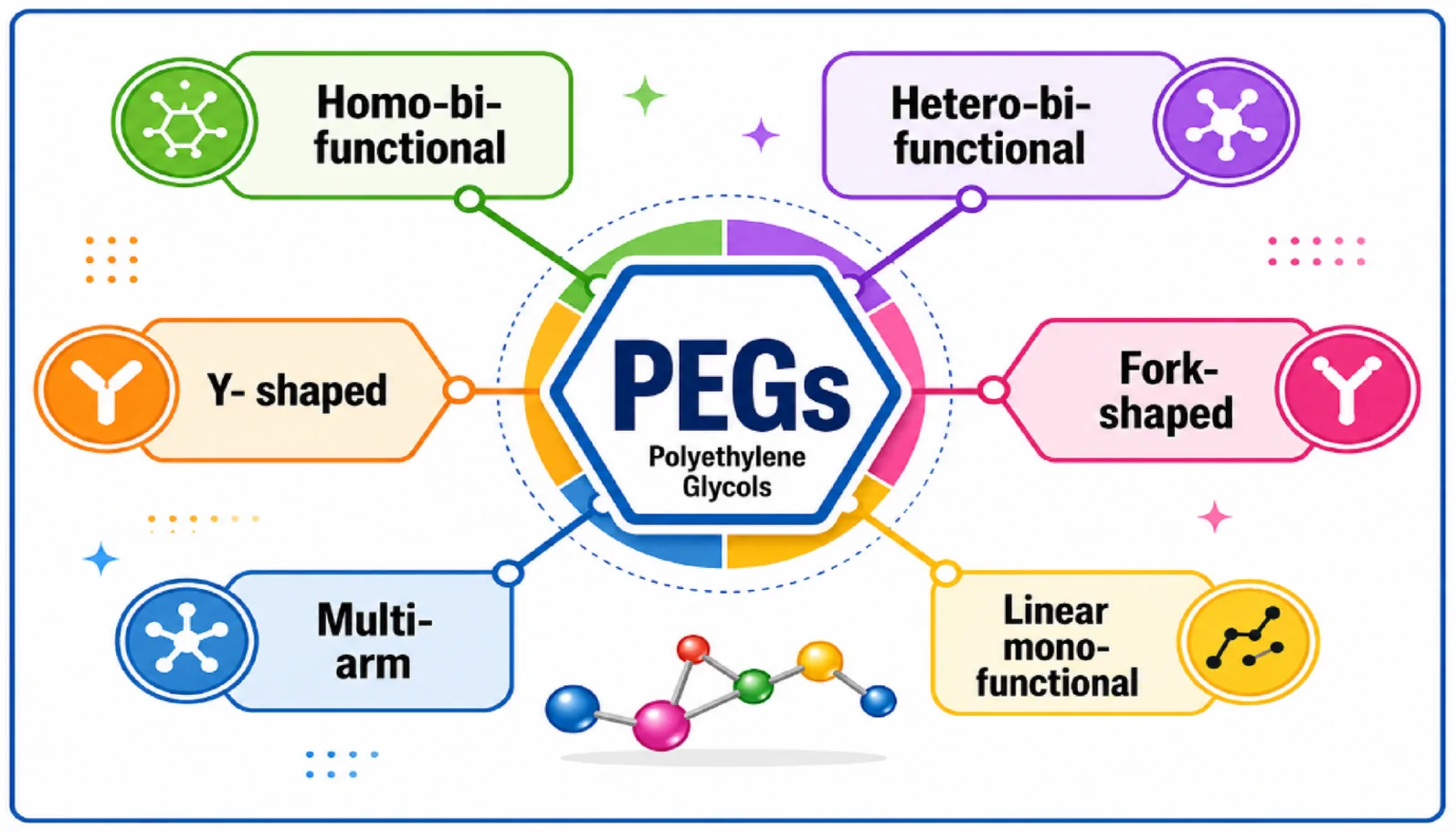
Polyethylene glycol (PEG) structures, showcasing a range of architectural designs such as homo-bifunctional, hetero-bifunctional, linear mono-functional, multi-arm, Y-shaped, and fork-shaped structures. The core PEG structure is linked to each variation to highlight the functional and design versatility of these structures for biomedical and materials engineering applications

**Fig. 14:**
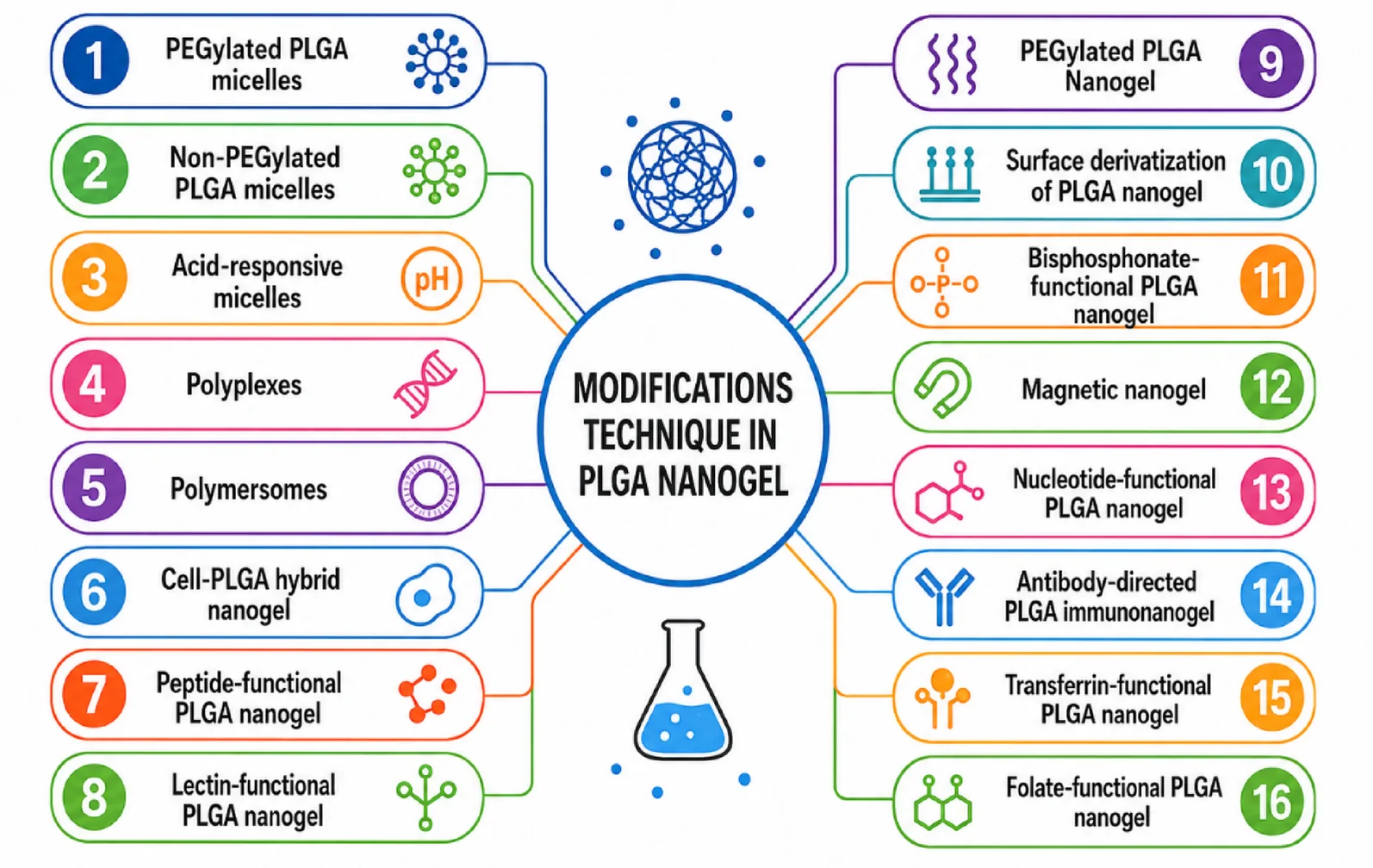
Schematic representation of PLGA nanogel modification strategies, showcasing various functionalization strategies such as micellar systems, polymer-based structures, and Bio functional nanogels, demonstrating their versatility to develop novel drug delivery systems

Polylactide-co-glycolide acid (PLGA)-based nanogels have been extensively investigated for the delivery of nucleic acids, proteins, vaccines, and therapeutic agents with the objective of achieving site-specific targeting. PLGA nanogels offer several advantages as nanocarriers, including sustained drug release, protection against drug degradation, and improved efficacy of cancer chemotherapy. In tumor tissues, abnormal vascularization and poor lymphatic drainage contribute to the enhanced permeability and retention (EPR) effect, which facilitates the preferential accumulation of nanocarriers within malignant tissues [126]. Enhanced permeation and retention (EPR) represent one of the most important mechanisms for effective tumor targeting [127]. PLGA nanogels can be administered through multiple routes, including oral, intravenous, and topical delivery [127]. Despite their promising therapeutic potential, PLGA nanogels exhibit certain limitations. To maximize the EPR effect, prolonged circulation and sustained release characteristics are required; however, premature elimination of nanocarriers due to opsonization frequently limits their effectiveness [128]. Consequently, PLGA-based nanogel and micellar systems have been developed to improve targeting efficiency and therapeutic outcomes [129]. Such limitations adversely affect biodistribution and ultimately reduce pharmacological activity and therapeutic efficacy [72]. Furthermore, compared with several other nanomaterials, PLGA possesses relatively few functional groups available for surface modification, making surface engineering more challenging [73]. Various strategies have been employed to improve the performance of PLGA nanogels, including PEGylation, polyion complexation, ligand conjugation, and surfactant coating. PEG-conjugated PLGA (PEG-PLGA) systems have been utilized to enhance the physicochemical and therapeutic properties of nanogels. Modification of terminal PEG groups with amino, carboxyl, or aldehyde functionalities can alter surface charge and improve targeting capabilities [130]. Polyethylene glycol derivatives are available in numerous configurations. Linear monofunctional PEG-coated nanogels containing amino or carboxyl groups on one side and methoxy or sugar moieties on the other exhibit enhanced stability and targeting efficiency. More recent developments include heterobifunctional PEG derivatives, Y-shaped PEG structures, and fork-shaped PEG derivatives (Fig. 13) [131]. PLGA has emerged as one of the most attractive biodegradable polymers for the encapsulation and controlled delivery of phytochemicals and used in skin cancer therapy. Owing to its excellent biocompatibility, biodegradability, approval for biomedical applications, PLGA provides a suitable matrix for improving the stability and bioavailability of natural anticancer compounds. Several phytoconstituents, including curcumin, quercetin, resveratrol, berberine, epigallocatechin gallate, genistein, and aloe vera-derived bioactive compounds, possess remarkable antioxidant, anti-inflammatory, antiproliferative, and pro-apoptotic properties [78]. However, their clinical application is hindered by poor aqueous solubility, rapid metabolism, and limited bioavailability. Encapsulation within PLGA-based nanogels can overcome these challenges by protecting phytochemicals from degradation and providing sustained and controlled release [79]. PLGA-based herbal nanogels are particularly advantageous for the treatment of melanoma and non-melanoma skin cancers because their nanoscale dimensions facilitate enhanced penetration and accumulation within tumor tissues through the EPR effect [80]. In addition, surface modification through PEGylation and ligand conjugation can further improve tumor selectivity and prolong systemic circulation, thereby increasing the therapeutic efficacy of herbal compounds while minimizing adverse effects on healthy tissues [81]. Topically administered PLGA herbal nanogels have also shown promise in improving the permeation of phytochemicals across the skin barrier and maintaining prolonged retention at the tumor site [82]. These properties are particularly beneficial for localized treatment of skin cancers, where sustained exposure to natural anticancer agents can enhance therapeutic outcomes. Consequently, PLGA-based herbal nanogels represent a versatile and promising platform that combines the advantages of biodegradable polymeric systems with the pharmacological potential of phytochemicals. Such formulations may contribute significantly to the development of safer, targeted, and more effective therapies for melanoma and other skin malignancies [131]. Polyethylene glycol (PEG) is available in numerous different forms, as shown in Fig. 14. Linear monofunctional PEG-coated nanogels consisting of amine or carboxy on one surface and sugar or methoxy on the other side have enhanced stability and targeting properties. Some of the recent advances in the field of PLGA-based nanogels are hetero-bi-functional PEG derivatives, Y-shaped bifurcated PEG, and fork-shaped PEG derivatives (Fig. 14) [131].

PLGA nanoparticles may provide a sustained release of antigens that have been encapsulated over a period of time. The longer durations that antigens are released could lead to the development of stronger immune reactions in vaccination. The economic and cost-effectiveness perspective of using smaller dosages of these particles may be Favorable [132].

### pH-triggered nanogels and polymeric nanocarriers

**Fig. 15:**
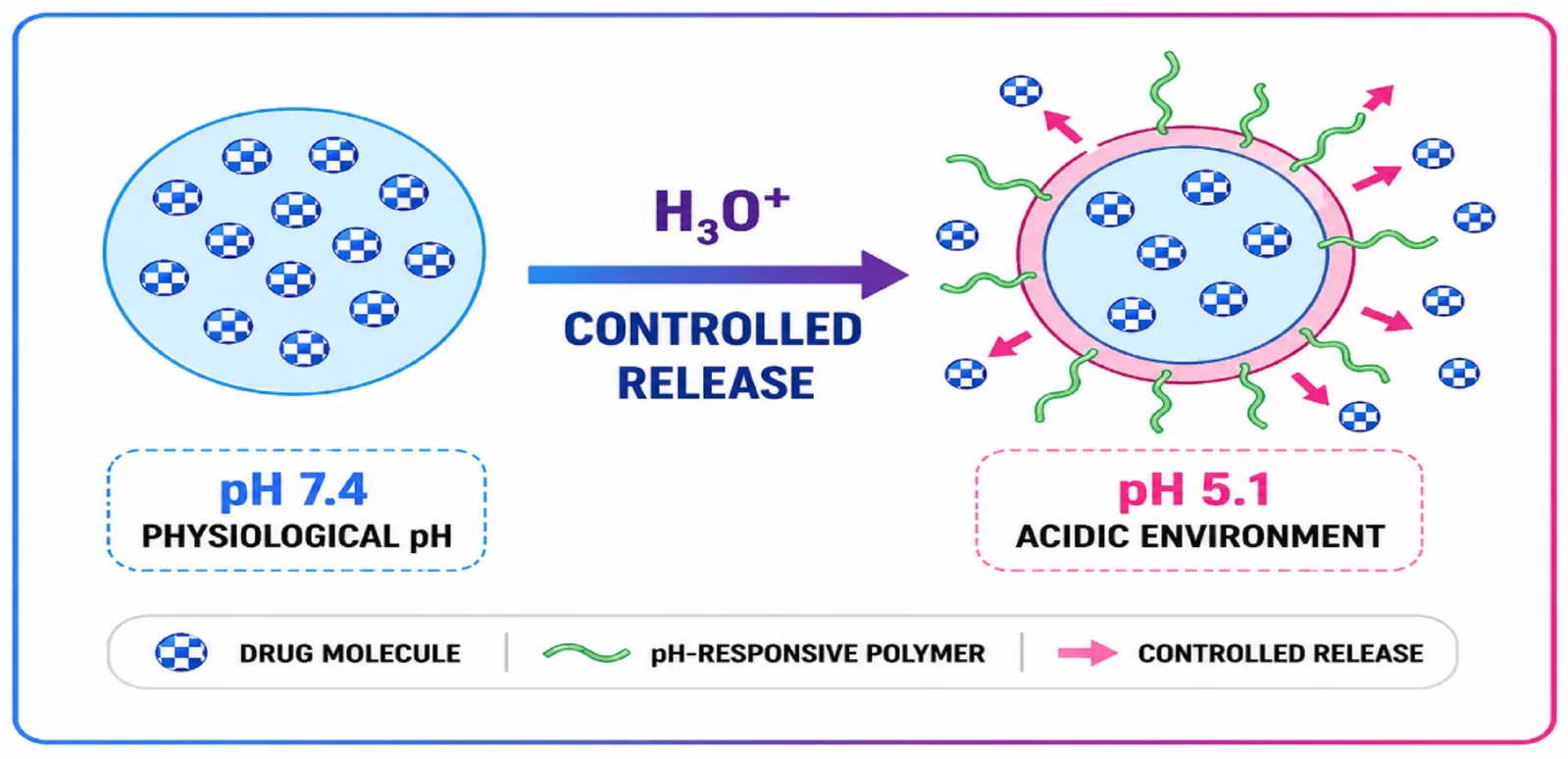
Schematic representation of controlled release from nanogels in response to pH

Micelles made of pH-responsive diblock copolymers have shown promise as an avenue for the conveyance of drugs, according to the investigation [133]. Additionally, several alternative pharmaceutical delivery methods have been created. Amphiphilic polymers connected by hydrazones or acetals form pH-dependent micelle structures or dendrimers that have cancer treatment potential [134]. This is now accomplished by conjugating the medicines with a pH-sensitive molecule. Furthermore, the medication is anticipated to be released at the cancerous area at a rate significantly higher than in the adjacent normal tissue structures, which maintain a physiological pH level of around 7.4 [135]. Consequently, one would expect chemotherapeutic drugs that are delivered via these systems to have lower adverse effects than could be significantly worse in some cases [136]. The polymer backbone or side chains should contain acid-labile or degradable functional groups to enable the erosion of the nanospheres at the surface or in bulk. Therefore, leads to the release of pH-sensitive medicine [137]. The poly (ortho esters) produces nanospheres with numerous desirable properties that make them useful as drug delivery vehicles [138]. Among them is a). PH-sensitive medication release b). Besides biocompatibility, c). Poor cytotoxicity [139]. Besides being minimally cytotoxic and highly cellular-absorbing, nanogel possesses a host of positive attributes that render it an appropriate drug delivery vehicle. These properties are pH-sensitive dissolution and drug release. The resulting pH susceptibility might serve as an advantage for medication distribution, as the polymeric molecule nanogel appears to be dissolved more quickly in a comparatively acidic buffered environment (pH 5.1) than in a neutral pH solution (pH 7.4 (Fig. 15) [140].

Nano- or microspheres of medications encapsulated in pH-sensitive polymers have been reported to be developed to target either antigen-presenting cells (APCs) or cancer cells. 14–Deblock pH-sensitive copolymer micelles have also demonstrated striking potential as drug delivery vehicles [141]. Various cancer indications have been developed with similar drug delivery systems. These techniques include the coupling of pharmaceuticals to amphiphilic polymers via a pH-cleaving bonding agent, such as hydrazone or acetal, to create pH-dependent micelle structures or the aforementioned dendrimers [142]. To enhance the effectiveness of current-generation cancer treatments, pH-assisted drug-containing innovative nanospheres could potentially be employed to focus on and carry out the drugs either directly to malignancies or through them, since the cancerous interstitial layer has a recognized acidic pH (5.5–6.5) compared with healthy tissues [142]. Furthermore, the medication is anticipated to be released at the site of cancer at a much higher rate than in adjacent normal tissues, which have a physiological pH of around 7.4. Consequently, it is expected that chemotherapy medication delivered via these systems would exhibit fewer adverse effects, although it could prove substantial in certain cases [143]. To exhibit controlled-release properties under acidic conditions, polymeric-based nanospheres or microspheres must have acid-dependent or degradation-resistant functional groups embedded within their underlying structure or as side chains. These functional categories facilitate interface or mass eroding of nanospheres, resulting in acid-sensitive drug release [144]. Table 4: Approaches for Minimizing Surfactant Toxicity in Topical Herbal Nanogels: Implications for Clinical Translation in Skin Cancer Treatment.

**Table 4:** Strategies to Mitigate Surfactant Toxicity in Topical Herbal Nanogel Formulations for Clinical Translation in Skin Cancer Therapy

Challenge/Issue	Effect on Skin Barrier and Clinical Translation	Strategy to Limit Surfactant Toxicity	Mechanism/Advantage	Ref
High surfactant concentration in inverse micellar emulsion and reverse micellar methods	Disruption of stratum corneum lipids, irritation, erythema, and increased trans epidermal water loss (TEWL)	Use surfactants below the critical micelle concentration (CMC) or irritation threshold	Preserves skin barrier integrity and minimizes lipid extraction	[179]
Use of ionic surfactants (e.g., SDS, CTAB)	Cytotoxicity and inflammatory responses	Replace with biocompatible non-ionic surfactants such as polysorbates, poloxamers, lecithin, and sucrose esters	Reduces irritation and enhances biocompatibility	[180]
Residual surfactants after synthesis	Chronic skin exposure and toxicity	Employ purification methods such as dialysis, ultrafiltration, centrifugation, and repeated washing	Removes unbound surfactants and improves safety	[181]
Surfactant-induced barrier damage	Increased permeability and skin sensitivity	Incorporate barrier-repair excipients such as ceramides, hyaluronic acid, glycerol, and chitosan	Restores hydration and barrier function	[182]
Local inflammatory reactions	Reduced patient compliance and therapeutic efficacy	Incorporate anti-inflammatory phytochemicals such as curcumin, aloe vera, and green tea polyphenols	Mitigates inflammation and oxidative stress	[183]
Dependence on surfactant-intensive methods	Limited scalability and regulatory hurdles	Utilize low-surfactant or surfactant-free approaches including ionic gelation, self-assembly, microfluidics, and radiation crosslinking	Improves clinical translation and manufacturing feasibility	[184]
Uncontrolled surfactant release	Prolonged skin exposure and irritation	Encapsulate surfactants within biodegradable matrices or employ stimuli-responsive systems	Reduces direct surfactant–skin interactions	[185]
Insufficient safety evaluation	Difficulty in regulatory approval	Perform skin irritation tests, TEWL studies, cytocompatibility assays, and in vivo dermatological assessments	Facilitates regulatory acceptance and clinical application	[186]

### Non-toxic biodegradable nanogels

In order to investigate gene function and to carry out gene therapy, it is essential to have the capability of transferring genetic material into cells, both in vitro and in vivo. In the past decade, the sphere has experienced a significant level of development. In order to identify the most suitable approach to providing a safe, secure, and target-specific gene delivery [145], one must first establish the optimal approach. Due to their ability to be used as novel therapeutic delivery vehicles in living cells, tissues, and organs, colloidal nanogel carriers have received significant interest. These networks cross-linked polymeric structures offer a core-shell structure, which is ideal in chemical engineering and in the encapsulation of materials in the core [146]. In addition to their minimal toxicity, nanogels are an excellent alternative carrier for the conveyance and spontaneous delivery of biologic molecules. Because nanogels are biodegradable, it is easy to entrap things within the core and release materials. Notably, nanogels encapsulating substantial genetic material molecules show significant agglomeration. Nanogel delivery techniques, on the other hand, are able to effectively incorporate siRNA. The estimated value of innovative nanomaterials is an important consideration in their prospective biological applications [147]. Nanogels between 70 and 90 nanometers are claimed to exhibit the highest gene expressions [148]. Smaller nanogels could potentially be more effective in translation than giant ones. Genome expression in nanogel-containing vectors may be improved by adding biocompatible-based moieties that are novel to cationic-based nanogels produced using the reversible additive-fragmentation chain transfer polymerisation method [149], leading to boosts in the dimensions, surface composition, and overall performance of the vectors. Lead to render it achievable to demonstrate a greater degree of control over the surface chemistry, dimensions, and gene transcription of vectors based on nanogel compounds. Table 5. Comprehensive Toxicological Evaluation and Safety Assessment Framework for Herbal Nanogel Formulations in Skin Cancer Therapy. Bhuchar et al. (2012) provided a description of the fabrication and characteristics of temperature-sensitive biodegradable cationic nanogels, as well as enhanced the expression of genes in Hep G2 cells [150]. They also noted that the nanogels were heat-resistant. The microscope techniques that were utilized to characterize the small repertoire of nanogels were scanning electron microscopy (SEM), transmission electron microscopy (TEM), and dynamic light scattering (DLS), which are examples of molecular imaging techniques. They conducted research that has just been revealed on the manufacture of degradation-resistant thermoresponsive, poly (2-methacryloyloxyethyl phosphorylcholine), poly-(MPC)-based nanogels to encapsulate and release proteins [151]. The further investigation served as a foundation for the rational development of the nanogel-containing platforms. Complexing genetic material with compostable nanogels that are responsive to pH as well as temperature is the sixteenth stage. The capacity of the nanogels, in particular, to consolidate plasmid genetic material is an essential condition for the development of a technique of delivering genetic material that is capable of functioning.

**Table 5:** Toxicological Concerns and Safety Assessment Framework for Herbal Nanogel Formulations in Skin Cancer Therapy

Toxicological Aspect	Herbal Drug-Related Concerns	Nanogel-Associated Concerns	Recommended Assessment Methods	Translational Implications	Key References
Long-term dermal safety	Skin irritation, allergic dermatitis, phytochemical sensitization	Local inflammation, barrier disruption, prolonged skin retention	Dermal irritation and sensitization studies, repeated-dose dermal toxicity, histopathology	Critical for chronic topical use safety	[164, 165, 166]
Nanoparticle accumulation and biodistribution	Altered pharmacokinetics of phytochemicals	Organ accumulation (skin, liver, spleen, kidneys), incomplete clearance	Biodistribution studies, pharmacokinetics, imaging (fluorescence/ICP-MS)	Essential for chronic toxicity risk assessment	[167, 168, 169]
Immunotoxicity	Herbal-induced hypersensitivity or immunomodulation	Cytokine release, complement activation, immune dysregulation	Cytokine profiling, immunophenotyping, complement assays	Important for predicting immune-related adverse effects	[170, 171, 172]
Oxidative stress and cellular toxicity	Pro-oxidant effects of phytochemicals at high doses	ROS generation, mitochondrial dysfunction, apoptosis	ROS assays, mitochondrial assays, DNA damage studies	Key mechanism of nano–bio interaction toxicity	[173, 174, 175]
Biodegradation-associated toxicity	Toxic herbal metabolites or reactive intermediates	Toxic degradation products of polymeric nanogels	Degradation kinetics, metabolite toxicity studies	Important for long-term safety evaluation	[176, 177]
Genotoxicity and mutagenicity	DNA damage from certain herbal constituents or contaminants	Nanoparticle-induced chromosomal abnormalities	Ames test, comet assay, micronucleus assay	Required for regulatory approval	[178, 179, 180]
Systemic toxicity	Hepatic, renal toxicity, herb–drug interactions	Off-target organ accumulation after absorption	Biochemical, hematological, histopathological studies	Ensures systemic safety evaluation	[181, 182, 183]
Stability-related toxicity	Degradation of phytoconstituents, loss of potency	Aggregation, leakage, instability of nanogel matrix	Stability studies, HPLC, particle size/zeta potential analysis	Ensures product consistency and shelf-life	[184, 185]
Batch-to-batch variability	Variability in plant source, season, extraction process	Variability in encapsulation efficiency and release profile	HPLC fingerprinting, LC-MS, QbD approaches	Critical for GMP compliance	[186, 187, 188]
Herb–nanocarrier interactions	Chemical incompatibility, altered bioactivity	Changes in drug loading/release behavior	FTIR, DSC, XRD, molecular modeling	Impacts therapeutic consistency	[189, 190]
Regulatory toxicological requirements	Lack of standardized herbal safety protocols	Nanomedicine-specific regulatory gaps	FDA/EMA/ICH-compliant toxicity testing	Required for clinical translation	[191, 192]
Translational safety challenges	Limited clinical validation of herbal nanomedicines	Uncertain long-term human safety data	Phase I–III clinical safety monitoring	Determines clinical feasibility	[193, 194]

### Bifunctional polymeric nanogels

Their superior stability and drug loading characteristics, in addition to the fact that they release drugs at different rates depending on environmental factors like temperature and pH, redox mechanisms, enzymes, and other biological stimuli, have motivated much interest in the development of stimuli-stimulated nanogels that can serve as functional drug delivery vehicles [152]. Some of the representative methods that are employed in the preparation of smart colloidal assemblies are inverse microemulsion polymerization [153], cross-linking of preformed polymeric micelles [98, 99] or vesicles (also known as polymersomes) [154], and one-step ring-opening polymerization. Such assemblies are generally made of cross-linked cores or shells of hydrophilic polymeric networks or core-shell architectures. The zwitterionic nanogels that were developed by Ganie et al. are biodegradable and multipurpose; they were able to include fluorescently labelled dextran into the gel for use as a medicinal product design, and Fe3O4 nanoparticles into the gel for use as an MR-based imaging probe [155]. Utilising the contrary microemulsion-based polymerisation of carboxylesterase methacrylate with l-cystine bis(acrylamide) as a disulphide (redox-sensitive) cross-linked component was the method that was used to accomplish [156]. It was stated that Kameshwar et al. successfully managed to synthesise two polymeric graft copolymers that had acrylic acid (AAc) and 2-methacryloylethyl acrylate (MEA) as components as the backbone. Additionally, grafting materials (B) PNIPAAm and monomethoxy (ethylene glycol) (mPEG) were described as having been synthesised. There is a graphical representation of the chemical configurations, chemical compositions, and average molecular weights of the graft copolymers that may be found in the figure [157].

## Emerging clinical trials involving nanogels

Nanogels have attracted considerable interest as multifunctional drug delivery platforms owing to their tunable physicochemical properties, high drug-loading capacity, and ability to enable controlled and targeted drug release. These characteristics have stimulated increasing efforts toward their clinical translation across a broad range of malignancies [158]. However, despite encouraging preclinical findings, the clinical development of nanogel-based therapeutics remains relatively limited, and currently available evidence should be interpreted with caution. Importantly, most ongoing clinical investigations involve nanotechnology-enabled formulations rather than herbal nanogels specifically designed for skin cancer. Several clinical studies have explored nanocarrier-mediated delivery of conventional chemotherapeutic agents for solid tumors with the aim of enhancing intratumoral drug accumulation and reducing systemic toxicity [159]. Clinical trials such as NCT03532538 and NCT03167051 have evaluated the delivery of doxorubicin and paclitaxel in patients with advanced pancreatic cancer and non-small cell lung cancer, respectively [160]. Although these studies highlight the potential of nanotechnology-assisted chemotherapy, their findings cannot be directly extrapolated to herbal nanogel formulations or to cutaneous malignancies because of differences in formulation composition, tumor biology, and therapeutic objectives. In haematological malignancies, nanotechnology-based approaches have also been investigated for the delivery of agents such as daunorubicin and vincristine. Clinical studies including NCT03116326 and NCT02751598 have focused on acute myeloid leukaemia and relapsed or refractory acute lymphoblastic leukaemia [161]. While these investigations demonstrate the broader applicability of nanocarrier systems in oncology, the long-term safety, pharmacokinetic characteristics, and comparative therapeutic advantages of nanogel-based formulations remain to be fully established. Targeted nanocarrier systems functionalized with tumor-specific ligands or antibodies have further expanded the scope of precision medicine. Clinical studies such as NCT03653572 and NCT03466727 have investigated antibody-mediated drug delivery strategies in ovarian cancer and HER2/neu-positive breast cancer, respectively [162]. Nevertheless, challenges associated with manufacturing complexity, batch-to-batch reproducibility, immunogenicity, and regulatory approval continue to impede their widespread clinical implementation. In addition, nanotechnology platforms are increasingly being explored in cancer immunotherapy for the delivery of immune-modulating agents and vaccines. Clinical investigations including NCT03685466 and NCT03989096 have evaluated their potential in non-small cell lung cancer and glioblastoma [163]. Although these approaches represent promising therapeutic avenues, evidence regarding their long-term efficacy and safety remains limited, and further studies are required to clarify their translational relevance. Overall, current clinical investigations provide valuable insights into the potential of nanotechnology-enabled drug delivery systems in oncology. However, there is a notable paucity of clinically validated studies specifically addressing herbal nanogel formulations for skin cancer. Therefore, the translational promise of these systems remains largely supported by preclinical evidence, underscoring the need for well-designed clinical studies, standardized manufacturing processes, comprehensive safety assessments, and regulatory frameworks to facilitate their progression from laboratory research to clinical application. Table 6: Comparative Summary of Recent Clinical Studies and Translational Outcomes of Nanotechnology-Enabled Drug Delivery Systems in Oncology (2021–2026) Table 7: Comparative Summary of Herbal Nanogel Formulations in Skin Cancer Therapy: Physicochemical Features, Anticancer Activity, Toxicological Profile, and Translational Potential.

**Table 6:** Recent Clinical Investigations and Translational Status of Nanotechnology-Based Drug Delivery Systems in Cancer Therapy (2021–2026)

Clinical Trial Identifier	Cancer Type	Therapeutic Strategy	Payload/Drug	Translational Significance	Major Limitations	Reference
NCT03532538	Advanced pancreatic cancer	Nanocarrier-assisted chemotherapy	Doxorubicin	Demonstrates the feasibility of nanotechnology-mediated chemotherapy and improved drug localization.	Not specific to herbal nanogels or skin cancer; limited clinical evidence available.	[160]
NCT03167051	Non-small cell lung cancer	Nanocarrier-mediated drug delivery	Paclitaxel	Supports the use of nanocarriers to improve pharmacokinetics and therapeutic efficacy in solid tumors.	Translational relevance to skin cancer remains uncertain.	[160]
NCT03116326	Acute myeloid leukaemia	Nanotechnology-enabled chemotherapy	Daunorubicin	Highlights the broader applicability of nanocarrier systems in hematological malignancies.	Long-term safety and comparative efficacy require further investigation.	[161]
NCT02751598	Relapsed/refractory acute lymphoblastic leukaemia	Targeted drug delivery approach	Vincristine	Suggests the potential of nanotechnology to enhance drug delivery and reduce systemic toxicity.	Lack of evidence regarding applicability to skin cancer and herbal nanogels.	[161]
NCT03653572	Ovarian cancer	Antibody-functionalized targeted delivery	Drug-loaded targeted formulation	Illustrates advances in precision medicine and selective tumor targeting.	Manufacturing complexity and immunogenicity concerns remain unresolved.	[162]
NCT03466727	HER2/neu-positive breast cancer	Ligand-mediated targeted therapy	Targeted anticancer agents	Demonstrates the promise of targeted nanoplatforms in improving tumor specificity.	Reproducibility and regulatory challenges hinder widespread clinical translation.	[162]
NCT03685466	Non-small cell lung cancer	Nanotechnology-assisted immunotherapy	Immune checkpoint modulators	Indicates the expanding role of nanotechnology in cancer immunotherapy.	Long-term efficacy and safety data are limited.	[163]
NCT03989096	Glioblastoma	Vaccine-based nanotechnology approach	Immunomodulatory agents	Represents emerging applications of nanotechnology in immunotherapy.	Clinical benefits and translational outcomes remain to be fully established.	[163]

HER2, human epidermal growth factor receptor 2. Importantly, although these clinical studies highlight the growing translational interest in nanotechnology-enabled drug delivery systems, none of the currently available trials specifically evaluate herbal nanogel formulations for skin cancer, emphasizing the substantial gap between preclinical promise and clinical translation

**Table 7:** Comprehensive Comparative Summary of Herbal Nanogel Formulations for Skin Cancer Therapy: Physicochemical Characteristics, Biological Performance, Safety, and Translational Status

Herbal Compound	Plant Source	Nanogel Composition/Polymer	Preparation Method	Particle Size (nm)	Zeta Potential (mV)	Encapsulation Efficiency (%)	Drug Loading (%)	Release Profile	Skin Cancer Model (In Vitro/In Vivo)	Molecular Mechanism/Target Pathway	Therapeutic Outcomes	Toxicity/Safety Profile	Clinical Status	Regulatory Considerations	Translational Limitations	Ref
Curcumin	*Curcuma longa*	Chitosan–alginate nanogel	Ionic gelation	120–250	+ 15 to + 35	75–95	10–25	Sustained, pH-responsive	A375 melanoma; murine melanoma models	PI3K/Akt inhibition, apoptosis induction, ROS generation	Reduced tumor growth and enhanced cellular uptake	Low cytotoxicity to normal cells; good biocompatibility	Preclinical	Requires standardization and GMP compliance	Poor scalability and batch variability	[164]
Resveratrol	*Vitis vinifera*	Hyaluronic acid nanogel	Emulsion crosslinking	100–200	−10 to − 25	70–90	8–20	Controlled release	SK-MEL-28, B16F10 models	NF-κB suppression, caspase activation	Increased apoptosis and reduced metastasis	Minimal systemic toxicity	Preclinical	Limited regulatory guidance for herbal nanomedicines	Low bioavailability and stability	[165]
Quercetin	Fruits and vegetables	Chitosan nanogel	Ionic gelation	150–300	+ 20 to + 40	65–90	5–15	Sustained release	A431 skin carcinoma	MAPK modulation and antioxidant activity	Inhibited proliferation and angiogenesis	Biocompatible with low hemolysis	Preclinical	Requires toxicity standardization	Manufacturing challenges	[166]
EGCG	*Camellia sinensis*	Gelatin nanogel	Self-assembly	80–180	−15 to − 30	70–92	8–18	pH-sensitive release	Melanoma models	ROS-mediated apoptosis and anti-inflammatory effects	Enhanced therapeutic efficacy	Low skin irritation	Experimental	Limited clinical evidence	Rapid degradation	[167]
Berberine	*Berberis vulgaris*	Poloxamer nanogel	Thermosensitive gelation	100–250	+ 10 to + 25	75–93	10–20	Sustained release	B16F10 melanoma	AMPK activation and cell cycle arrest	Tumor regression	Favorable safety profile	Preclinical	Herbal product approval complexity	Limited pharmacokinetic data	[168]
Apigenin	Parsley, chamomile	Carbopol nanogel	Solvent evaporation	120–220	−5 to − 20	70–88	5–12	Controlled release	SCC cell lines	p53 activation and apoptosis	Reduced tumor progression	Good dermal compatibility	Experimental	Insufficient regulatory frameworks	Scale-up difficulties	[169]
Genistein	Soybean	PEG-based nanogel	Chemical crosslinking	100–200	−10 to − 25	72–90	8–16	Prolonged release	Melanoma models	Tyrosine kinase inhibition	Improved anticancer efficacy	Minimal organ toxicity	Preclinical	Requires long-term safety evaluation	Lack of clinical trials	[170]
Thymoquinone	*Nigella sativa*	Chitosan nanogel	Ionic gelation	150–250	+ 18 to + 35	80–95	10–22	pH-responsive release	A375 melanoma	NF-κB inhibition and oxidative stress modulation	Enhanced apoptosis and reduced tumor burden	Biocompatible and safe	Preclinical	Need for standardized manufacturing	Regulatory uncertainty	[171]

EGCG, epigallocatechin gallate; ROS, reactive oxygen species; GMP, good manufacturing practice; PEG, polyethylene glycol

## Emerging controversies and knowledge gaps in herbal nanogel-based skin cancer therapy

Although herbal nanogel formulations have attracted increasing attention as a strategy for localized and targeted skin cancer therapy, several controversies continue to hinder their widespread acceptance. A major concern is the discrepancy between the impressive anticancer activity observed in preclinical studies and the scarcity of successful clinical translation. Most of the study show the promising antiproliferative, pro-apoptotic, and anti-inflammatory effects; however, these findings are predominantly based on in vitro cell lines and murine tumor models. Such models often fail to accurately reproduce the heterogeneity and immunological complexity of human skin cancers. Another challenge lies in the intrinsic variability of herbal bioactive compounds. Factors including geographical origin, harvesting conditions, extraction methods, and phytochemical composition can significantly influence biological activity, resulting in inconsistent therapeutic outcomes [179]. Moreover, uncertainty remains regarding the exact mechanisms responsible for the synergistic interactions between phytoconstituents and nanogel matrices. While numerous studies describe enhanced efficacy, mechanistic evidence explaining cellular uptake, immune modulation, and tumor microenvironment interactions remains limited. Furthermore, concerns have been raised regarding nanoparticle accumulation, long-term toxicity, and environmental impact following large-scale production. Consequently, future investigations should emphasize mechanistic studies, clinically relevant disease models, and standardized evaluation protocols to bridge existing knowledge gaps. 6.1 Translational Barriers and Clinical Challenges [180]: Despite encouraging preclinical findings, the translation of herbal nanogel formulations into clinically approved therapies remains challenging. One of the principal barriers is the limited availability of pharmacokinetic, biodistribution, and long-term toxicological data. Recent reports indicate that fewer than 10% of nanomedicine candidates successfully progress from preclinical development to clinical trials, highlighting the complexity of translating laboratory findings into therapeutic products [181]. Tumor heterogeneity and patient-to-patient variability further complicate treatment efficacy. Differences in skin thickness, vascularization, pH, oxidative stress levels, and immune responses may substantially influence drug penetration and release behavior. Additionally, the enhanced permeability and retention (EPR) effect, which is frequently cited as the basis for nanoparticle accumulation, has shown considerable variability in clinical settings and remains a subject of ongoing debate [182]. Another important challenge is the limited number of clinical studies evaluating herbal nanomedicines. Most currently available evidence originates from experimental models, with insufficient data concerning dose optimization, adverse effects, and long-term safety in humans. Addressing these limitations will require multidisciplinary collaboration among pharmaceutical scientists, oncologists, dermatologists, and clinical researchers. 6.2 Regulatory Considerations for Herbal Nanomedicines [183]: The regulatory evaluation of herbal nanogel formulations represents a complex task because these systems combine the challenges associated with botanical products and nanotechnology-based drug delivery systems. Regulatory agencies such as the FDA, EMA, and ICH have established general guidelines for nanomedicines; however, there remains no universally harmonized framework specifically addressing herbal nanotechnology products [184]. Recent regulatory discussions have emphasized the importance of implementing Quality-by-Design (QbD) approaches, comprehensive physicochemical characterization, impurity profiling, and robust safety assessments [78]. Critical quality attributes including particle size distribution, zeta potential, encapsulation efficiency, and release kinetics must be carefully controlled to ensure product consistency. Moreover, the complexity of phytochemical mixtures poses additional challenges for standardization and quality assurance [185]. Regulatory authorities increasingly advocate the use of advanced analytical techniques, including HPLC, LC-MS/MS, and metabolomics-based fingerprinting, to ensure reproducibility and batch consistency [186]. The development of dedicated international regulatory frameworks for herbal nanomedicines is expected to become a major priority in the coming years. 6.3 Manufacturing Scalability and Quality Assurance [187]: Large-scale production remains one of the most significant barriers preventing the commercialization of herbal nanogel formulations. Although laboratory-scale synthesis methods have demonstrated excellent therapeutic performance, their translation into industrial manufacturing often encounters challenges related to process reproducibility, stability, and cost-effectiveness ]188]. Variability in herbal raw materials and extraction procedures can result in batch-to-batch inconsistencies, affecting particle characteristics and therapeutic performance. Furthermore, conventional preparation techniques may be difficult to reproduce on a commercial scale. Recent advances in microfluidics, continuous manufacturing, and Quality-by-Design approaches have emerged as promising strategies for overcoming these limitations. Artificial intelligence-assisted process optimization and machine learning-based predictive models are also gaining attention for improving formulation robustness and manufacturing efficiency [189]. Such technologies may facilitate the transition from small-scale production to commercially viable manufacturing processes while ensuring regulatory compliance and product quality. 6.4 Reproducibility and Standardization Issues [190]: Poor reproducibility has emerged as a major concern in nanomedicine research. Variations in plant species, extraction solvents, geographical sources, storage conditions, and nanoparticle preparation techniques can lead to substantial differences in biological activity [191]. Consequently, results obtained by different research groups often exhibit limited comparability. Another important issue is the lack of standardized protocols for characterization and biological evaluation. Differences in methods used for determining particle size, drug loading, release kinetics, cytotoxicity, and stability frequently result in conflicting findings [192]. Recent recommendations from international nanomedicine communities have highlighted the need for standardized reporting guidelines and minimum characterization requirements. Advanced analytical tools such as dynamic light scattering, electron microscopy, Fourier-transform infrared spectroscopy, differential scanning calorimetry, and omics-based approaches are increasingly being employed to enhance reproducibility and improve quality assessment. Establishing harmonized protocols and reference standards will be essential for accelerating clinical translation [193]. 6.5 Commercialization Challenges and Market Perspectives: Despite the remarkable growth of the global nanomedicine sector, the successful commercialization of herbal nanogel formulations remains limited. Recent market analysts estimate that the global nanomedicine market was valued at approximately USD 190.6 billion in 2023 and is projected to exceed USD 410 billion by 2030, driven primarily by advances in oncology, targeted therapeutics, and precision medicine [194]. Nevertheless, herbal nanomedicines represent only a small segment of this rapidly expanding market, reflecting the substantial gap between academic innovation and clinical implementation. One of the major impediments to commercialization is the complexity associated with translating laboratory-scale formulations into industrially viable products. Although numerous herbal nanogel systems have demonstrated promising anticancer activity in preclinical studies, only a limited number of nanomedicines have progressed to regulatory approval. Recent studies have identified uncontrolled scale-up processes, high manufacturing costs, formulation complexity, and insufficient understanding of nano–bio interactions as major barriers to clinical translation [195]. Manufacturing-related challenges are particularly critical for herbal nanogel formulations. Variability in phytochemical composition arising from differences in plant sources, cultivation conditions, extraction procedures, and storage conditions can lead to significant batch-to-batch inconsistencies [195]. Furthermore, maintaining particle size distribution, encapsulation efficiency, sterility, and long-term stability during large-scale production remains difficult. Consequently, regulatory authorities increasingly emphasize the implementation of Quality-by-Design (QbD) principles, critical quality attributes (CQAs), and process analytical technologies to ensure reproducibility and product quality [196]. Recent reports suggest that QbD-based approaches, coupled with machine learning and artificial intelligence-assisted optimization, may improve process robustness and facilitate commercial manufacturing [196]. Regulatory uncertainty represents another important challenge. Herbal nanogels occupy a unique position at the interface of botanical medicines and nanotechnology-based drug delivery systems, resulting in ambiguity regarding their regulatory classification. Although the United States Food and Drug Administration (FDA) and the European Medicines Agency (EMA) have established general frameworks for nanomedicines, dedicated guidelines specifically addressing herbal nanogel formulations are currently lacking. Consequently, developers are required to provide extensive physicochemical characterization, stability studies, toxicological assessments, pharmacokinetic data, and evidence of clinical efficacy, which substantially increases development costs and timelines [197]. Intellectual property protection further complicates commercialization. Unlike synthetic drugs, naturally derived phytoconstituents are often difficult to patent, thereby limiting market exclusivity and reducing the attractiveness of these products to industrial investors. Moreover, reimbursement uncertainties, stringent regulatory requirements, and the relatively limited availability of robust clinical data discourage private-sector investment. Recent commercialization analyses have therefore advocated a transition from a material-centred approach toward a “commercialization-by-design” strategy, in which market demand, regulatory expectations, health economics, and clinical utility are considered during the early stages of product development [198]. Another important issue is the high attrition rate observed during nanomedicine development. Despite decades of research, only a small proportion of Nano formulations successfully reach clinical use, and several promising candidates have failed during late-stage clinical evaluation owing to inadequate therapeutic efficacy or unfavourable pharmacokinetic behavior. These experiences highlight the necessity for more predictive preclinical models and comprehensive translational assessment. Nevertheless, recent advances in continuous manufacturing technologies, microfluidics, artificial intelligence-assisted formulation optimization, and Quality-by-Design methodologies are expected to improve scalability and reduce manufacturing variability. Strategic collaborations among academia, pharmaceutical industries, biotechnology companies, regulatory agencies, and public funding organizations are likely to play a pivotal role in accelerating commercialization [199]. In addition, the development of harmonized international guidelines, standardized toxicological evaluation protocols, and GMP-compliant manufacturing platforms will be essential for bridging the gap between preclinical promise and commercial success [200]. Therefore, future progress in herbal nanogel-based skin cancer therapeutics will depend not only on demonstrating biological efficacy but also on overcoming the interconnected challenges of manufacturing, regulatory approval, intellectual property protection, and market accessibility. Table 8: Evidence-Based Comparative Evaluation of Herbal Nanogels and Existing Skin Cancer Therapies: Clinical Effectiveness, Nanocarrier Platforms, Limitations, and Translational Status.

**Table 8:** Structured Comparative Evaluation of Herbal Nanogels with Current Clinical and Nanotechnology-Based Skin Cancer Therapies: Mechanisms, Clinical Evidence, Limitations, and Translational Status

Therapy / Platform	Mechanism of Action	Clinical Evidence (Recent Status)	Key Advantages	Major Limitations	Translational Status	Comparative Position vs. Herbal Nanogels	Key Recent References
Herbal Nanogels	Phytochemical-induced apoptosis, ROS modulation, anti-inflammatory and anti-proliferative effects with controlled topical delivery	Predominantly preclinical (in vitro & in vivo skin tumor models); no established clinical approval	Biocompatibility, natural antioxidant activity, localized delivery, reduced systemic exposure	Lack of clinical trials, phytochemical variability, instability, unclear pharmacokinetics, regulatory uncertainty	Early-stage translational research	Promising but unvalidated clinically; inferior to approved therapies in evidence strength	[201]
Topical Imiquimod	TLR7 activation → cytokine-mediated immune response against tumor cells	FDA-approved for actinic keratosis and superficial BCC	Strong clinical efficacy, immune activation, non-invasive	Local inflammation, erythema, variable response rates	Established clinical standard	More reliable and validated than herbal nanogels	[202]
5-Fluorouracil (5-FU)	Antimetabolite inhibiting thymidylate synthase → DNA synthesis blockade	Widely used first-line topical chemotherapy	High efficacy, cost-effective, well-established	Severe skin irritation, cytotoxicity, poor selectivity	Clinically established	Higher toxicity but far more clinically validated	[203]
Photodynamic Therapy (PDT)	Light-activated photosensitizer generates ROS → tumor cell death	Clinically used for non-melanoma skin cancer; growing nanocarrier-enhanced trials	Site-specific, good cosmetic outcomes, repeatable	Pain, limited penetration depth, photosensitivity	Established clinical modality	Comparable localized approach but more standardized outcomes	[204]
Immunotherapy (PD-1/PD-L1 inhibitors)	Immune checkpoint blockade → T-cell activation	Standard for advanced melanoma; multiple FDA approvals	High survival benefit in metastatic disease	Immune-related adverse events, high cost	Fully established systemic therapy	Superior systemic efficacy compared to nanogels	[205]
Targeted Therapy (BRAF/MEK inhibitors)	Inhibition of MAPK signaling pathway	Approved for BRAF-mutant melanoma	Rapid tumor response rates	Drug resistance, relapse, toxicity	Clinically established	Molecular precision therapy beyond nanogel capability	[206]
Liposomes	Phospholipid vesicles improving drug solubility and delivery	Widely used clinically; several FDA-approved formulations	High biocompatibility, improved pharmacokinetics	Limited skin penetration, leakage issues	Advanced clinical nanocarrier	More mature and clinically validated than herbal nanogels	[207]
Solid Lipid Nanoparticles (SLNs)	Solid lipid matrix enabling controlled release	Extensive preclinical + early clinical dermatological applications	High stability, controlled release, low toxicity	Limited drug loading, polymorphic transitions	Translationally advanced	More standardized and reproducible than herbal nanogels	[208]
Polymeric Micelles	Self-assembled amphiphilic polymers enhancing solubility	Clinical-stage evaluation in anticancer drug delivery	Improved solubility, tumor targeting potential	In vivo instability, premature drug release	Advanced translational stage	More clinically mature than herbal nanogels	[75]

ROS, reactive oxygen species; BCC, basal cell carcinoma; PDT, photodynamic therapy; FDA, United States Food and Drug Administration

## Conclusion

Herbal nanogels represent an emerging and promising drug delivery platform that integrates the therapeutic potential of natural bioactive compounds with advances in nanotechnology-based delivery systems for skin cancer management. Preclinical studies suggest that these formulations may enhance dermal penetration, improve solubility and stability of phytoconstituents, and enable localized and sustained drug release at tumor sites. Such properties may help overcome several limitations associated with conventional herbal preparations, including poor bioavailability, rapid degradation, and lack of site specificity. Experimental evidence further indicates that herbal nanogel systems can modulate key biological processes involved in skin cancer progression, including tumor cell proliferation, oxidative stress pathways, and apoptosis. In addition, their potential for combination with established treatment modalities such as photodynamic therapy and immunotherapy has been explored in preclinical settings, suggesting possible synergistic effects. Moreover, the incorporation of stimuli-responsive strategies—including pH-responsive, enzyme-responsive, receptor-targeted, and microenvironment-sensitive nanogels—offers additional opportunities for achieving precision delivery of phytochemicals within the unique skin cancer microenvironment characterized by weak acidity, elevated enzyme activity, and complex tumor heterogeneity. Such approaches may enhance therapeutic specificity while minimizing off-target toxicity. However, despite these encouraging findings, the clinical translation of herbal nanogels remains limited. Most available evidence is derived from in vitro and animal studies, and robust clinical trials evaluating their safety, pharmacokinetics, and therapeutic efficacy in humans are still lacking. Furthermore, the presence of the stratum corneum barrier, interpatient variability in tumor microenvironmental characteristics, and limited penetration into deeper skin lesions pose additional challenges to the effective clinical implementation of topical and transdermal herbal nanogel systems. Important challenges such as formulation standardization, long-term safety, large-scale manufacturing, regulatory approval, and batch-to-batch reproducibility continue to restrict their clinical adoption. The inherent variability of herbal extracts, including differences in phytochemical composition arising from plant source, cultivation conditions, and extraction methods, further complicates quality control and regulatory standardization of herbal nanomedicines. Therefore, while herbal nanogels hold significant potential as next-generation nanomedicine platforms for skin cancer therapy, their clinical relevance remains to be fully established. Future research should also focus on patient-specific nanomedicine approaches, scalable manufacturing technologies, and standardized evaluation protocols to facilitate successful bench-to-bedside translation. Integration of artificial intelligence, advanced biomaterials, and personalized medicine strategies may further accelerate the clinical development of herbal nanogel-based therapeutics for skin cancer. Future research should prioritize rigorous clinical validation, mechanistic studies, and translational frameworks to bridge the gap between experimental promise and real-world therapeutic application. A cautious and evidence-driven approach will be essential to determine their true role in future oncological practice. Collectively, although substantial scientific progress has been achieved, herbal nanogels remain largely at the preclinical stage, underscoring the need for multidisciplinary collaborations among pharmaceutical scientists, oncologists, material engineers, and regulatory agencies to establish their safety, efficacy, and long-term clinical utility in skin cancer management.

## Data Availability

No datasets were generated or analysed during the current study.
